# A denoising autoencoder–enhanced ResNet 50 framework for explainable brain tumor classification using MRI

**DOI:** 10.3389/fonc.2026.1806663

**Published:** 2026-08-14

**Authors:** Magbool Alelyani, Sultan Alamri, Ahmad Joman Alghamdi, Sahal Alotaibi, Nahla L. Faizo, Adel Alshehri, Ahlam A. Alzaidi, Abdullah A. Asiri, Arwa Baeshen, Njoud Aldusary, Batil Alonazi

**Affiliations:** 1 Department of Radiological Sciences, College of Applied Medical Science, King Khalid University, Abha, Saudi Arabia; 2 Department of Radiological Sciences, College of Applied Medical Sciences, Taif University, Taif, Saudi Arabia; 3 Radiological Sciences department, College of Applied Medical Sciences, Najran University, Najran, Saudi Arabia; 4 Radiological Sciences Department, College of Applied Medical Sciences, King Saud University, Riyadh, Saudi Arabia; 5 Radiologic Sciences Department, Faculty of Applied Medical Sciences, King Abdulaziz University, Jeddah, Saudi Arabia; 6 College of Medicine, Alfaisal University, Riyadh, Saudi Arabia

**Keywords:** brain tumors, deep learning, digital health, explainable artificial intelligence, MRI

## Abstract

Brain tumors are a major issue in neurological health where the timely and accurate diagnosis is critical in the effectiveness of the clinical intervention and the outcome of the patients. It is believed that magnetic resonance imaging (MRI) is the imaging modality that is predominantly used to evaluate brain tumors due to the fact that it has excellent soft-tissue contrast, however, the analysis of automated MRI is hindered by noise, inhomogeneity in intensities, inter-scanner variation, and even the visual similarity that is exhibited by different tumor subtypes. In order to overcome these challenges, the current research presents a powerful, understandable deep learning architecture to be used in the context of multi-classification of brain tumors using MRI scans. The proposed methodology is a denoising autoencoder (DAE) based image improvement step with a transfer-learning based ResNet-50 classifier. The DAE is trained without any supervision to suppress noise and emphasize salient anatomical structures before classification. The improved images then undergo refinement of a pretrained ResNet-50 network to label MRI scans with four clinically relevant classes. The experimental assessment of a publicly available benchmark dataset shows that the framework has a test accuracy of 98.93% and the precision, recall, and F1-scores are high in all classes. Furthermore, the gradient mapping technique (Grad-CAM) is used to provide visual explainability of the model predictions by highlighting image regions that influence the classification outcomes. These results show the potential of the proposed framework as an explainable AI-assisted decision-support approach for brain tumor MRI classification. Further validation using external and multi-center clinical datasets is required before clinical deployment.

## 1 Introduction

Brain tumors are among the most complex and life-threatening neurological disorders. They pose significant challenges for diagnosis and treatment worldwide. Recent global cancer statistics show that primary brain and central nervous system tumors remain a major cause of morbidity and mortality. Specifically, the malignant tumors such as gliomas exhibiting particularly poor prognosis and survival rates (1, 2). Therefore accurate and timely diagnosis is very essential for effective treatment planning, including surgical intervention, radiotherapy and chemotherapy. Magnetic Resonance Imaging (MRI) is widely regarded as the preferred imaging modality for brain tumor assessment. This is because of its superior soft-tissue contrast, non-invasive nature, and also its ability to provide detailed anatomical and pathological information (3). MRI enables clinicians to evaluate tumor location, morphology and tissue heterogeneity. However, manual interpretation of MRI scans is labor-intensive. It is also subject to inter-observer variability. Furthermore, MRI images are often affected by noise, intensity inhomogeneity, scanner-dependent variations and acquisition artifacts. This can degrade image quality and complicate both visual assessment and automated analysis (4, 5).

To assist radiologists and to improve diagnostic consistency, computer-aided diagnosis (CAD) systems have been extensively investigated in the literature. Early CAD approaches relied on handcrafted feature extraction methods. This includes texture descriptors, wavelet transforms, and intensity-based statistics, combined with conventional machine learning classifiers (6, 7). Although these methods give promising results. But their dependence on manual feature engineering limited their robustness and generalization capability across diverse datasets. The emergence of deep learning, particularly convolutional neural networks (CNNs), has significantly advanced medical image analysis. This is done by enabling automatic feature learning directly from image data (8). CNN-based models have achieved state-of-the-art performance in numerous medical imaging applications, including brain tumor classification and segmentation (9, 10). Advanced architectures such as DenseNet and EfficientNet have further improved classification performance through enhanced feature reuse and parameter efficiency (11, 12). Transfer learning has also become an effective method for addressing the limited availability of labeled medical imaging data. The Transfer learning is adapting those models which are pretrained on large-scale datasets such as ImageNet (13). Recent published studies have reported classification accuracies exceeding 95% for multiclass brain tumor MRI classification using architectures such as ResNet, DenseNet, EfficientNet, and transformer-based models (14–18).

Despite these advances, several challenges remain unaddressed. First, many existing studies apply deep learning models directly to raw or minimally processed MRI images. They did not adequately address image degradation and noise which are potentially affecting feature extraction and classification reliability. Second, high-performing deep learning models often operate as black-box systems with limited interpretability. This reduces clinician trust and hindering clinical adoption (19, 20). Third, many published papers emphasize predictive accuracy and they are giving less attention to robustness, transparency and explainability. While these are essential requirements for practical healthcare applications and digital health systems (21).

To address the interpretability challenge, explainable artificial intelligence (XAI) techniques have gained a lot of attention in medical imaging research. Recent published studies have shown that integrating explainability mechanisms with deep learning models can enhance transparency and can support clinical decision-making (22–24). Methods such as Grad-CAM can provide visual explanations by highlighting image regions that contribute most strongly to model predictions. Thus improving model interpretability and user confidence (25, 26). At the same time, image enhancement before classification remains an important topic. It is comparatively underexplored component of brain tumor analysis pipelines. Conventional denoising techniques such as median filtering and anisotropic diffusion rely on fixed parameters. They may not generalize well across heterogeneous MRI datasets (27). Consequently, there is a growing need for intelligent frameworks which are capable of simultaneously improving image quality, maintain high classification performance and provide interpretable diagnostic outputs.

Motivated by these observations in the published literature, this study proposes a two-stage deep learning framework. This framework combines a denoising autoencoder (DAE) with a transfer learning-based ResNet-50 classifier for multiclass brain tumor MRI classification. The DAE is employed to enhance image quality and to suppress noise before classification. The ResNet-50 network performs robust tumor recognition. To improve transparency, Grad-CAM is integrated into the framework to generate visual explanations of model predictions. By jointly addressing image enhancement, classification accuracy and interpretability, the proposed framework aims to provide an interpretable and explainable framework for AI-assisted brain tumor MRI classification and future decision-support applications.

The main contributions of this research study are listed below:

- A DAE-enhanced transfer-learning framework for brain tumor MRI classification that integrates learned image enhancement and deep feature extraction, with its effectiveness quantitatively validated through comprehensive ablation analysis.
- A comprehensive statistical validation protocol including five-fold cross-validation, confidence interval estimation, pooled ROC analysis, and robustness assessment to evaluate model reproducibility and generalization capability.
- A multi-method explainability framework using Grad-CAM, LIME and SHAP, supported by both qualitative visualization and quantitative evaluation metrics to improve transparency of model decision-making.

Collectively, these contributions provide a robust, interpretable, and statistically validated framework for automated brain tumor MRI classification and establish a foundation for future decision-support research.

## 2 Related work

Initial studies on brain tumor MRI classification were mainly done by manually extracting features with the use of standard machine learning classifiers. SVMs and k-NN classifiers were often utilized with texture-based descriptors, wavelet transforms, and intensity statistics, which delivered moderate classification results when operating under controlled settings (6, 7). Nevertheless, such methods were very sensitive to the design of features and could not hold much strength with respect to heterogeneous data. Convolutional neural networks represented a new medical image analysis paradigm. CNNs allow learning hierarchical features using image data, thereby hugely boosting results in brain tumor classification and segmentation (8–10). Further research investigated more general architectures, such as VGG, ResNet, and DenseNet, to better the level of feature discrimination, as well as to boost generalization (11, 24). Transfer learning has gained prominence especially in brain tumor MRI analysis because there is hardly any annotated data. Leveraging pretrained CNNs and fine-tuning has continued to be more successful in training than training all the way to the end, with enhanced convergence and robustness (13). Recent results based on pretrained ResNet, DenseNet, and EfficientNets have achieved classification rates of over 95 percent on multi-class brain tumor data (14–16, 28).

Still, a lot of available literature implements CNNs on the MRI images without paying specific attention to noise and intensity non-uniformity. The bias field effects and MRI artifacts may negatively influence deep feature extraction and decrease their robustness especially in multi-center or cross-dataset testing (5). Traditional preprocessing methods are based on hand-knifed parameters, and are usually not flexible between imaging protocols (27).

Models of autoencoders have been explored as data-driven versions of image denoising and enhancement. Originally proposed by Vincent et al. (29), denoising autoencoders are learned to generate clean images when fed corrupted ones by means of training. Layer-wise relevance propagation (LRP) has been used to explain CNN decisions in MRI-based diagnosis by producing relevance heatmaps (30). Later research had shown them to be useful in medical image denoising and feature enhancement (31). Nevertheless, relatively little is known as to combining denoising autoencoders with deep transfer learning classifiers to brain tumor MRI classification. Optimization-driven deep learning approaches have also been explored for brain tumor classification from MRI images. For example, Kumar and Mankame (32) proposed an optimization-driven deep convolutional neural network to improve classification performance by tuning network parameters. Although their method achieved promising accuracy, it relied on direct learning from raw MRI images without incorporating an explicit image enhancement or denoising stage. Moreover, the framework did not include explainability mechanisms to interpret model decisions, which limits its clinical transparency and applicability in real-world diagnostic settings. Rasheed et al. (33) proposed an MRI-based brain tumor classification framework that integrates image enhancement techniques with convolutional neural networks, achieving competitive performance on multi-class tumor classification tasks. Noreen et al. (34) investigated brain tumor MRI classification using fine-tuned deep convolutional models combined with an ensemble strategy, reporting improved performance over single-network approaches. Masood et al. (35) proposed a DenseNet-41–based Mask R-CNN framework for brain tumor recognition and classification from MRI images, achieving a classification accuracy of 98.34% on the Figshare dataset. Although their approach demonstrated strong performance, it relied on conventional preprocessing techniques and did not incorporate an explicit learned denoising mechanism or explainability analysis, which limits its robustness to MRI noise and clinical interpretability. Artificial intelligence and deep learning have demonstrated substantial potential for improving medical image interpretation and supporting data-driven healthcare applications (36).

The second significant drawback of diagnostic systems based on deep learning is the absence of interpretability. CNNs are also often said to be black-box, which makes them tricky to implement clinically and have regulatory acceptance (17). Grad-CAM, LIME and SHAP are some of the XAI methods that have been suggested to offer visualizations and enhance clarity (20, 29). Newer research also highlights the idea that explainability must be incorporated into the diagnostic pipeline and not a *post-hoc* analysis (20, 26).

Altogether, although recent literature demonstrates high classification rates in the context of brain tumor MRI analysis (14–16, 28), only a limited number of studies introduce a comprehensive framework that would optimally incorporate image enhancement, high classification rates, and explainability in accordance with the Digital Health needs. This disconnect inspires the given strategy.

Compared to published studies, this paper suggests a unified model that directly combines denoising autoencoder based image improvement, deep transfer learning with ResNet-50, and explainability with Grad-CAM. The proposed approach aims to bridge the gap between high-performance AI models and more interpretable decision-support frameworks.

## 3 Methodology

This section describes the dataset, preprocessing pipeline, denoising autoencoder design, classification architecture, system workflow and implementation details of the proposed framework. A flow chart of the entire system is given to add to the clarity and reproducibility. The hardware and software includes MATLAB R2025b, Deep Learning Toolbox, Windows 11, Intel Core Ultra 9 275HX processor, NVIDIA GeForce RTX 5090 Laptop GPU with 24 GB GDDR7 memory, 64 GB DDR5 RAM, and 2 TB NVMe SSD storage.

### 3.1 Dataset description

**Figure 1:**
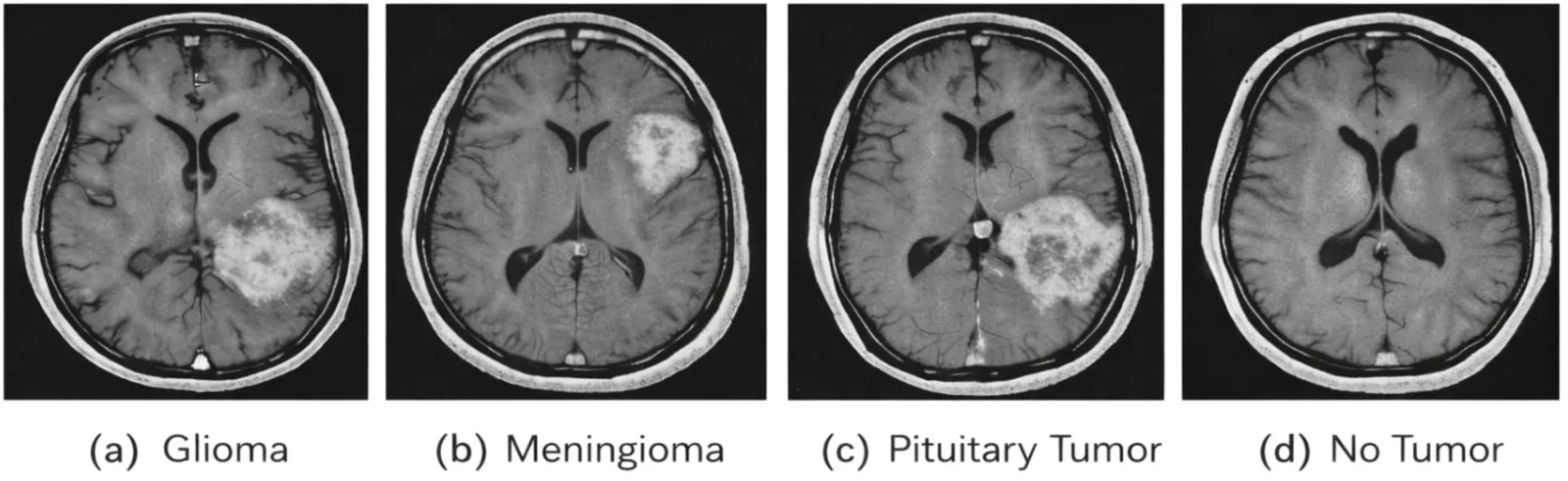
Sample MRI images of **(a)** glioma **(b)** meningioma **(c)** pituitary tumor and **(d)** no-tumor.

A brain MRI dataset is used to evaluate the proposed framework, which has been extensively used in recent published literature. The data is composed of T1 weighted contrast enhanced MRI scans of various subjects. It contains four clinically relevant classes. These types of tumors are typical intracranial tumors. They have different morphological appearances. They have irregular infiltrative patterns in gliomas and compact and well-defined structures in pituitary tumors. The original Kaggle dataset provides predefined training and testing subsets. **Table 1** is giving the statistical summary of the dataset. The validation images were derived from the original training subset during model development using stratified image-level random partitioning while preserving class distribution consistency. **Figure 1** shows the sample images. These sample images emphasize the visual heterogeneity and diagnostic complexity of the dataset. This visual heterogeneity highlights the necessity for robust image enhancement and discriminative feature learning.

**Table 1:** Statistical summary and class-wise distribution of the utilized Kaggle brain tumor MRI dataset.

Category	Training images	Validation images	Testing images	Total images
Glioma	1120	201	300	1621
Meningioma	1135	204	306	1645
Pituitary Tumor	1238	219	300	1757
No-Tumor	1362	233	405	2000
Total	4855	857	1311	7023

The test sample was made up of 1,311 MRI samples, thus ensuring that no test samples were seen during training. The hierarchical partitioning scheme is regularly used in medical imaging studies, as it provides an unbiased approximation of generalization performance as well as enables a strict hyperparameter tuning (Litjens et al., 2017).

### 3.2 Overview of the proposed framework

The proposed method follows a two-stage architecture comprising:

1. Image enhancement using a denoising autoencoder (DAE)
2. Multi-class classification using a transfer learning-based ResNet-50 network

**Figure 2:**
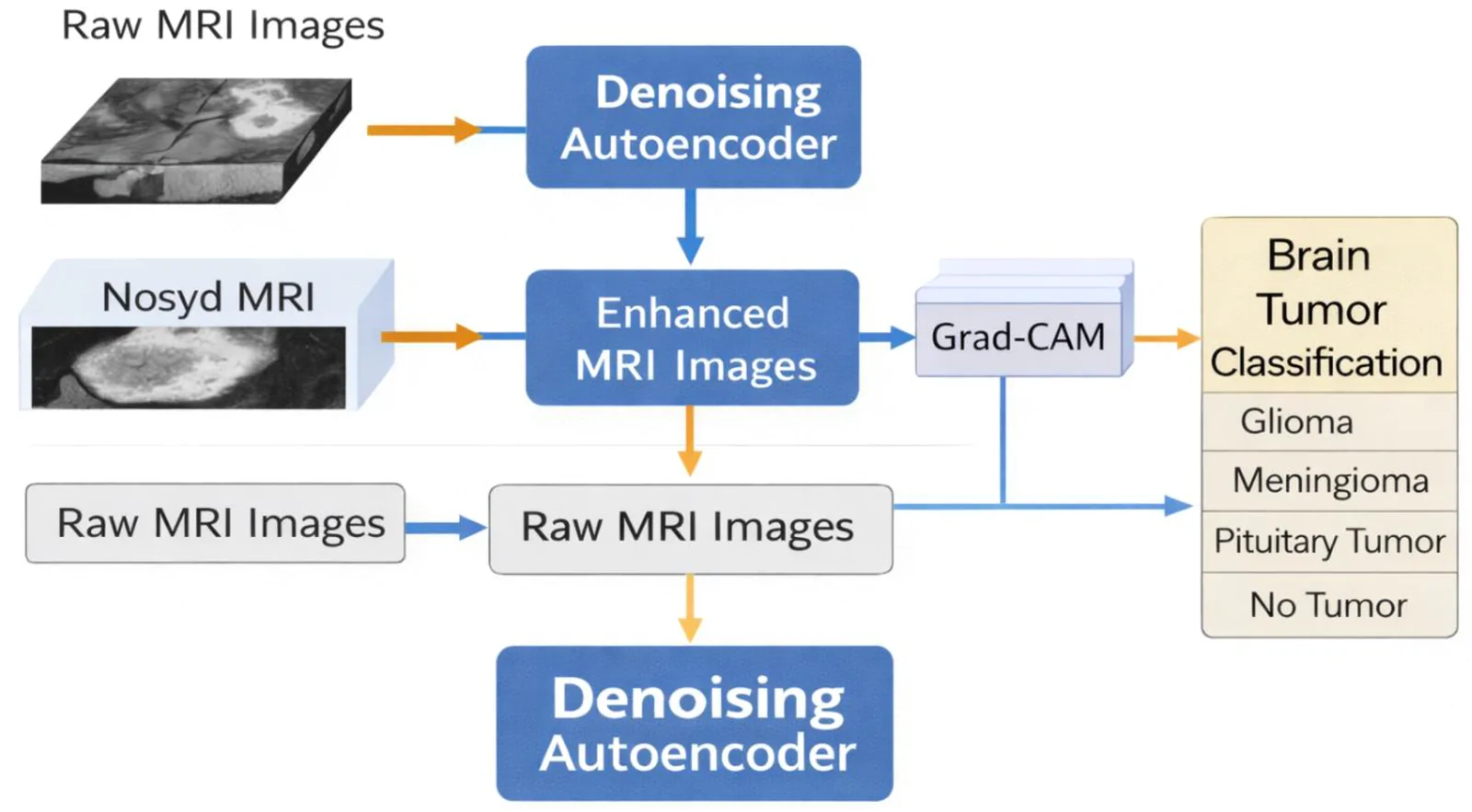
Conceptual framework of the proposed method.

The conceptual view of the framework is shown in **Figure 2**.

### 3.3 System architecture

**Figure 3:**
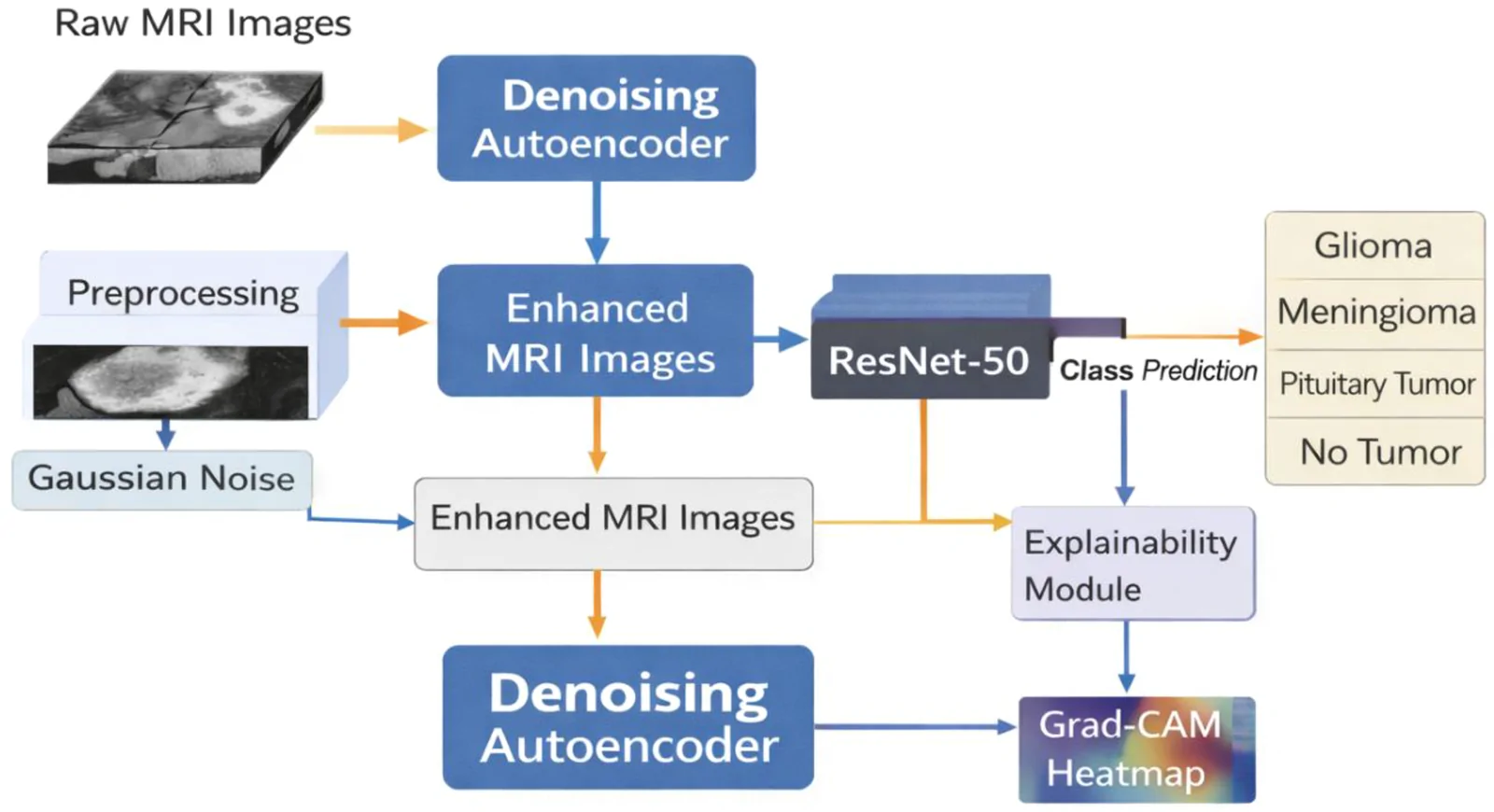
System architecture of the proposed framework.

**Figure 3** shows the entire system architecture. Raw MRI images are initially denoised with an auto encoder. The improved images are then fed into a ResNet-50 classifier that classifies the tumors. Explainability is achieved through Grad-CAM visualization of the final convolutional layers.

### 3.4 Preprocessing pipeline

**Figure 4:**
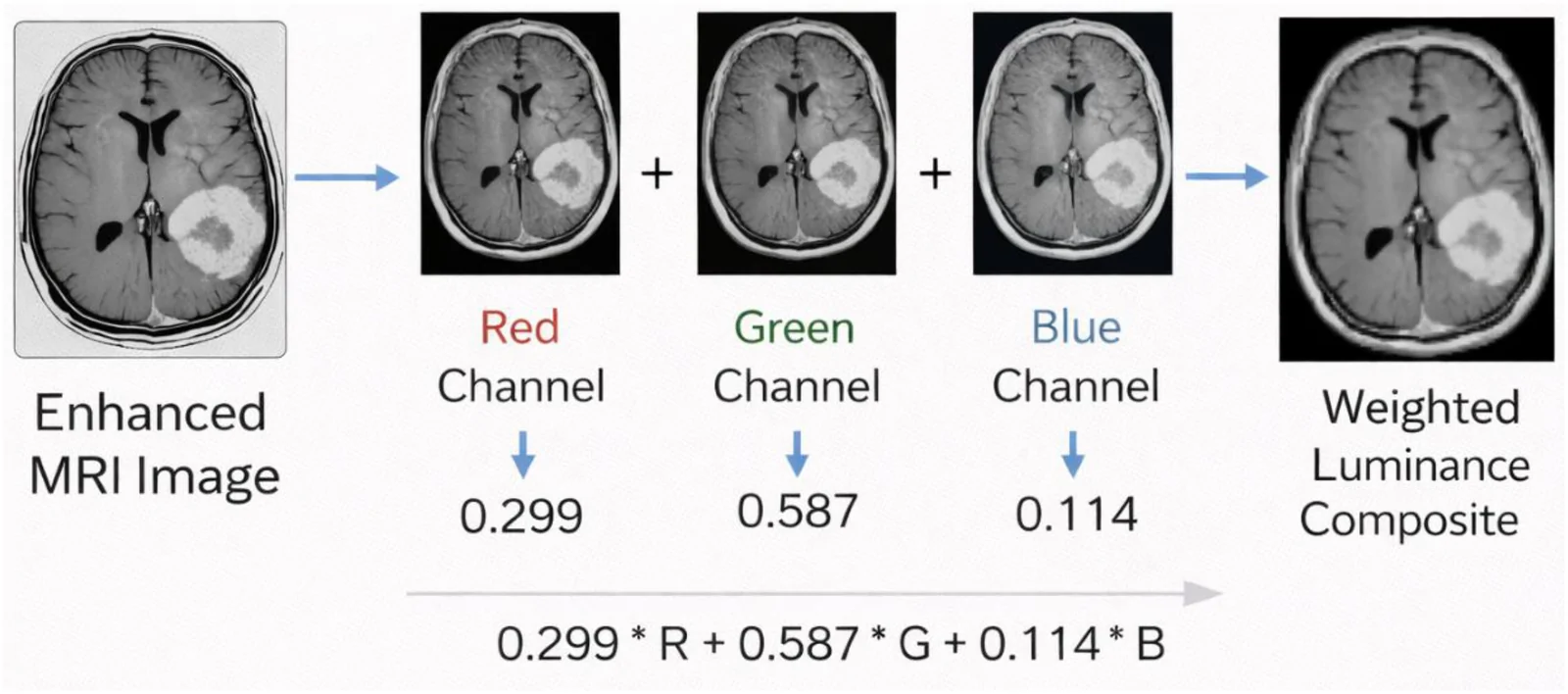
RGB-to-grayscale luminance conversion applied to the MRI dataset images.

Each MRI image was resized to 224 × 224 pixels to match the input dimensions of the ResNet-50 architecture. Pixel intensities were normalized to the range [0,1] to improve numerical stability during training. Although MRI scans are inherently grayscale, the publicly available Kaggle dataset used in this study stores the images in RGB image format. Since the three RGB channels contain nearly identical intensity information, so a weighted luminance transformation was applied to convert the images into a single-channel grayscale representation prior to denoising and classification. This preprocessing step reduces computational redundancy. While it preserve diagnostically relevant anatomical information. The weighted luminance conversion process is shown in **Figure 4**. Unlike conventional preprocessing methods that are based on fixed filtering operations, the proposed method subsequently employs a learned denoising autoencoder to adaptively suppress noise and to enhance image quality.

### 3.5 Denoising autoencoder

Let $X\in {\text{R}}^{H\times W}$ denote a clean, noise-free magnetic resonance (MR) image, where *H* and *W* represent the height and width of the image in pixels, respectively. Each element of *X* corresponds to the intensity value of a single pixel in the spatial domain. The noisy observation of the same image is represented by $\tilde{X}$, which is generated by corruption processes such as acquisition noise, scanner artifacts, or intensity non-uniformities commonly encountered in brain MRI.

The denoising autoencoder (DAE) consists of two primary components: an encoder and a decoder. The encoder function, denoted as $f_{\theta }(\cdot )$, maps the noisy input image $\tilde{X}$ into a compact latent representation *Z*. Here, $Z\in {\text{R}}^{d}$ is a lower-dimensional feature space that captures the most salient structural and intensity-related characteristics of the input image while suppressing noise. The parameter set θ represents the learnable weights and biases of the encoder network, optimized during training.

The decoder function $g_{\phi }(\cdot )$ reconstructs the enhanced image $\hat{X}$ from the latent representation Z. The reconstructed output $\hat{X}\in {\text{R}}^{H\times W}$ has the same spatial resolution as the original clean image and aims to preserve diagnostically relevant anatomical structures while reducing noise and contrast inconsistencies. The parameter set ϕ denotes the learnable parameters of the decoder network.

These encoder–decoder mappings are mathematically expressed as in **Equation 1**:

(1) $$Z=f_{\theta }(\tilde{X}),\hat{X}=g_{\phi }(Z)$$

To train the DAE, the reconstruction error between the clean reference image Xand the reconstructed output $\hat{X}$ is minimized using the mean squared error (MSE) loss function. The MSE quantifies the average squared difference between corresponding pixel intensities and is defined using **Equation 2**:

(2) $$L_{\text{DAE}}=\frac{1}{N}\overset{N}{\underset{i=1}{\sum }}∥X_{i}-{\hat{X}}_{i}{∥}^{2}$$

**Figure 5:**
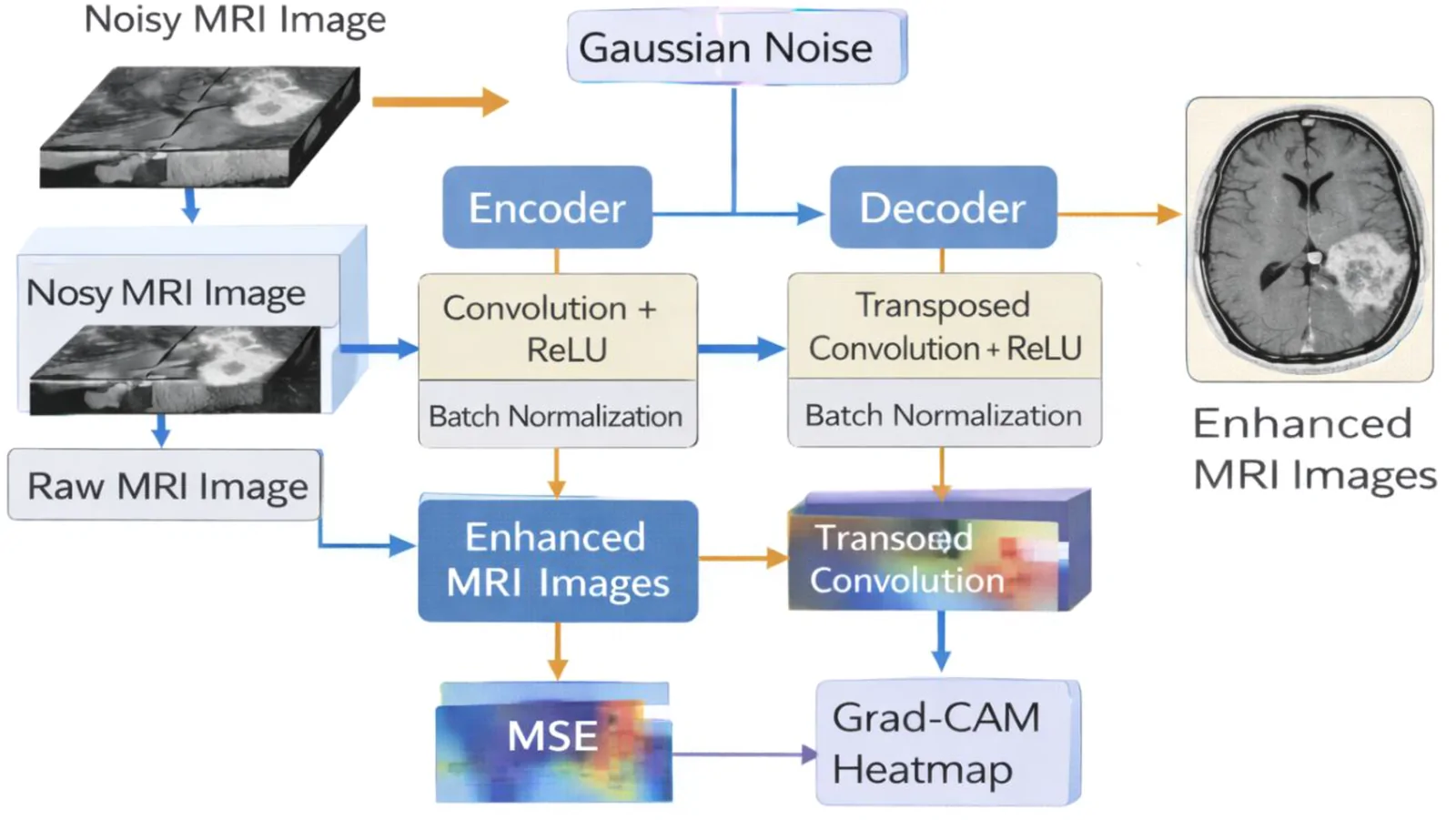
Architecture of the denoising autoencoder.

In this formulation, Ndenotes the total number of training samples, $X_{i}$represents the $i^{th}$clean MRI image in the dataset, and ${\hat{X}}_{i}$ is its corresponding reconstructed output produced by the DAE. The operator $∥\cdot {∥}^{2}$ denotes the squared Euclidean (ℓ_2_) norm computed over all pixel locations. Minimizing this loss encourages the network to learn a pixel-wise accurate reconstruction, thereby improving image clarity and enhancing tumor-relevant regions prior to classification. The block diagram of the denoising autoencoder is shown in **Figure 5**. The detailed architecture of the denoising autoencoder used for MRI image enhancement has been given in **Table 2**.

**Table 2:** Detailed architecture of the denoising autoencoder used for MRI image enhancement.

Stage	Layer type	Filter/kernel size	No. of filters	Stride/operation	Activation	Output size
Input	Image input layer	–	–	Input MRI image	–	224 × 224 × 1
Encoder-1	Convolution	3 × 3	32	Stride 1, same padding	ReLU	224 × 224 × 32
Encoder-1	Max pooling	2 × 2	–	Downsampling ×2	–	112 × 112 × 32
Encoder-2	Convolution	3 × 3	64	Stride 1, same padding	ReLU	112 × 112 × 64
Encoder-2	Max pooling	2 × 2	–	Downsampling ×2	–	56 × 56 × 64
Encoder-3	Convolution	3 × 3	128	Stride 1, same padding	ReLU	56 × 56 × 128
Latent Representation	Bottleneck feature map	–	128	Compact encoded representation	ReLU	56 × 56 × 128
Decoder-1	Transposed convolution/upsampling	3 × 3	64	Upsampling ×2	ReLU	112 × 112 × 64
Decoder-2	Transposed convolution/upsampling	3 × 3	32	Upsampling ×2	ReLU	224 × 224 × 32
Reconstruction	Convolution	3 × 3	1	Stride 1, same padding	Sigmoid	224 × 224 × 1
Output	Reconstructed enhanced MRI	–	–	Denoised image output	–	224 × 224 × 1

### 3.6 Classification network: ResNet-50

**Figure 6:**
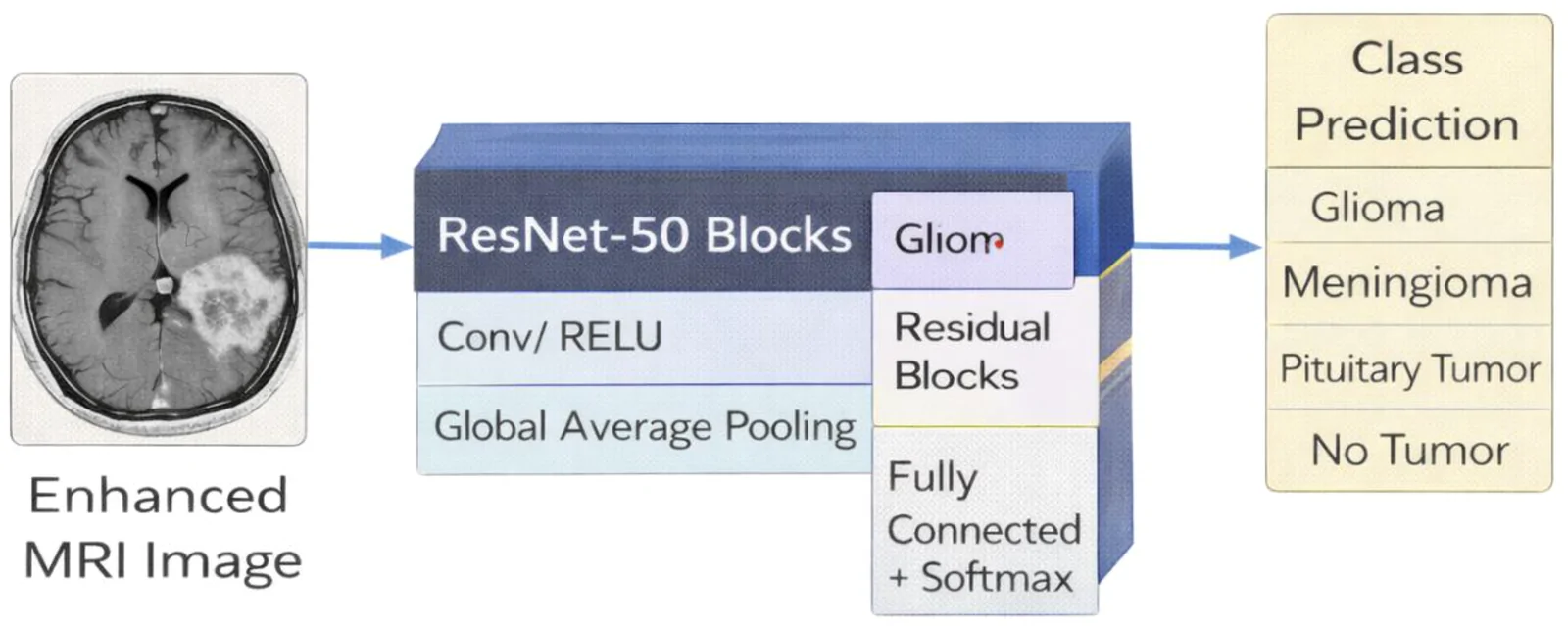
Architecture of the ResNet-50 classifier.

**Figure 7:**
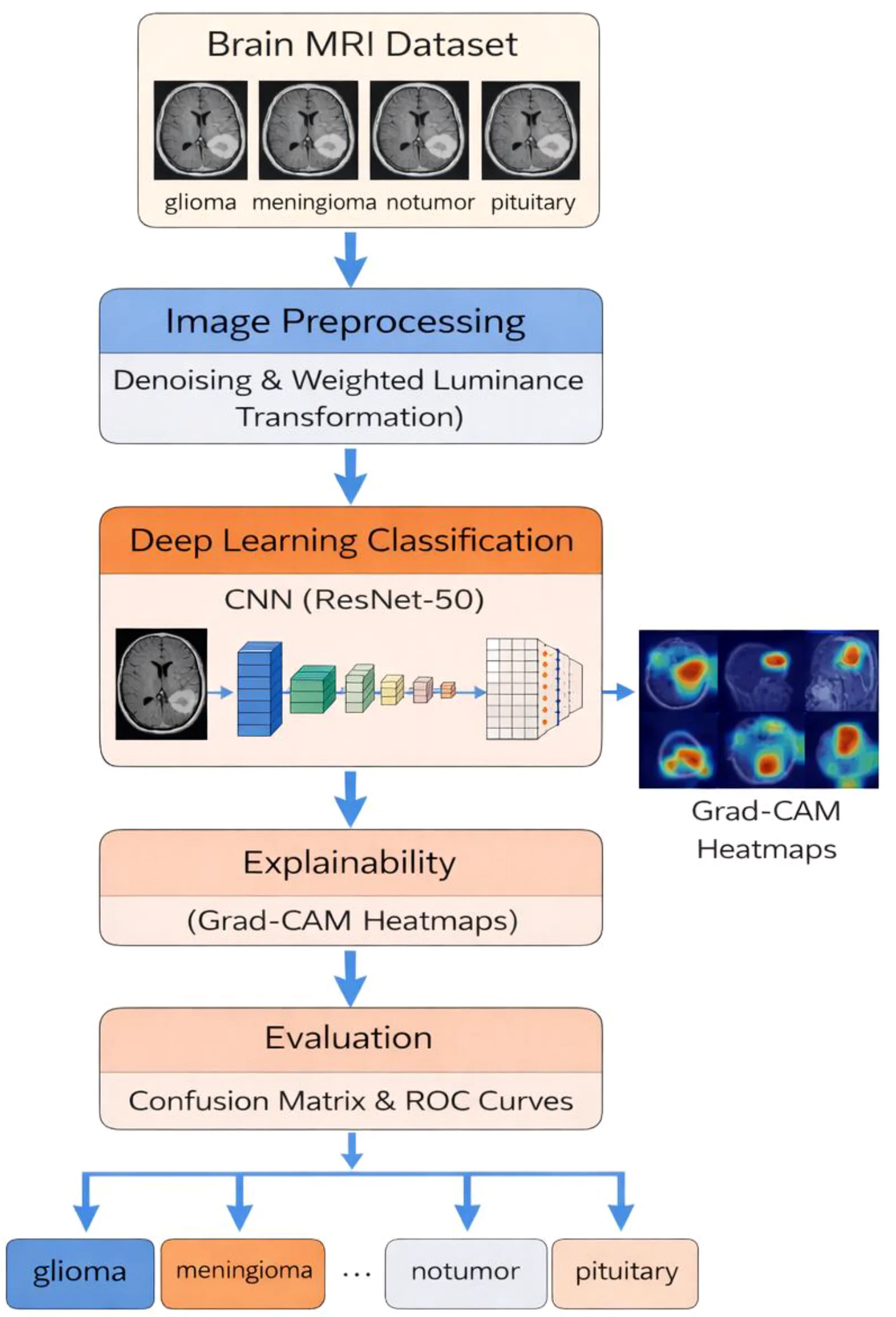
Flowchart of the proposed method.

For classification, a ResNet-50 network has been trained on the ImageNet database and is used through transfer learning. Deep networks can be trained effectively using residual connections that help alleviate the vanishing gradient problem. The last fully connected and softmax layer is changed with task-specific layers which reflect the four tumor classes. **Figure 6** illustrates the block diagram of the ResNet-50 based classification network. **Figure 7** provides an overview of the entire operational process of the proposed method. Detailed architecture and transfer-learning modifications of the proposed ResNet-50 classifier is given in **Table 3**.

**Table 3:** Detailed architecture and transfer-learning modifications of the proposed ResNet-50 classifier.

Stage	Layer/component	Configuration	Purpose	Activation/function	Output
Input	Image Input Layer	224 × 224 × 3 MRI image	Input preprocessing	Normalization	224 × 224 × 3
Backbone	Pretrained ResNet-50	50-layer deep residual CNN	Feature extraction	Residual learning	2048-D feature vector
Transfer Learning	Frozen Initial Layers	Early convolutional layers frozen	Retain generic visual features	—	Reduced trainable parameters
Feature Extraction	Residual Blocks	Deep residual connections	Hierarchical feature learning	ReLU	High-level tumor features
Modification-1	Fully Connected Layer	4 output neurons	Tumor classification	Linear	4-class logits
Modification-2	Softmax Layer	Probability normalization	Class probability estimation	Softmax	Class probabilities
Classification	Classification Output Layer	Cross-entropy loss	Final classification	Cross-entropy	Predicted tumor class
Optimization	Optimizer	Adam optimizer	Network training	Adaptive optimization	Optimized weights
Training Setting	Mini-batch Size	16	Stable GPU training	—	Batch learning
Training Setting	Initial Learning Rate	1 × 10^-4^	Controlled convergence	—	Optimized learning
Training Setting	Epochs	20 epochs	Model convergence	—	Trained classifier
Regularization	Data Augmentation	Rotation, reflection, translation	Improve generalization	—	Augmented MRI samples
Explainability	Grad-CAM/LIME/SHAP	*Post-hoc* explainability analysis	Interpret model decisions	Heatmap generation	Visual explanations

**Table 4** gives a summary of the main architectural settings of denoising auto encoder and ResNet-50 classifier. The training hyperparameters used in the experiments are summarized in **Table 5**, all the experiments were done in MATLAB via Deep Learning Toolbox. The training was done on a normal workstation environment. The entire pipeline, such as preprocessing, enhancement, classification, and explainability, is fully reproducible, thus facilitating transparency and repeatability.

**Table 4:** Model architecture parameters.

Component	Parameter	Value
DAE	Encoder layers	3 convolutional layers
DAE	Latent feature maps	128
DAE	Loss function	Mean Squared Error
ResNet-50	Total layers	50
ResNet-50	Trainable parameters	~25.6 million
ResNet-50	Output classes	4

**Table 5:** Training hyperparameters.

Parameter	Value
Optimizer	Adam
Initial learning rate	1 × 10^-4
Batch size	16
DAE epochs	15
Classification epochs	20
Data augmentation	Rotation, reflection, translation
Loss function (classifier)	Cross-entropy

In order to improve reproducibility, the additional implementation settings are also specified in this section. During DAE training, Gaussian corruption noise with a standard deviation of σ = 0.05 was added to the normalized grayscale MRI images. The pixel intensities were scaled to the range [0, 1]. The clean normalized images were used as reconstruction targets. The DAE was optimized using mean squared error loss. All experiments are conducted using a fixed random seed rng(42) to ensure reproducible data splitting, augmentation and training initialization. A fixed-epoch training strategy is adopted instead of early stopping to ensure identical training budgets across experiments. The DAE is trained for 15 epochs and the ResNet-50 classifier is trained for 20 epochs. Hyperparameters, including learning rate, mini-batch size, number of epochs, augmentation settings, and DAE noise level, were selected through preliminary validation-set monitoring by considering convergence behavior, validation accuracy, validation loss stability and computational feasibility.

## 4 Results and discussion

In order to fully test the proposed framework, some performance measures were used. To measure the overall classification performance, accuracy was employed and precision, recall and the F1 score were employed to measure the behavior of the class. Furthermore, confusion matrices and receiver operating characteristic (ROC) curves were created to describe dynamics of classification. The DAE-boosted ResNet-50 model reached an accuracy of 98.93% on the independent test set, which implies a strong generalization and shows that the combination of denoising-based enhancement with deep transfer learning provides a significant boost to the classification of brain MRI images.

**Figure 8:**
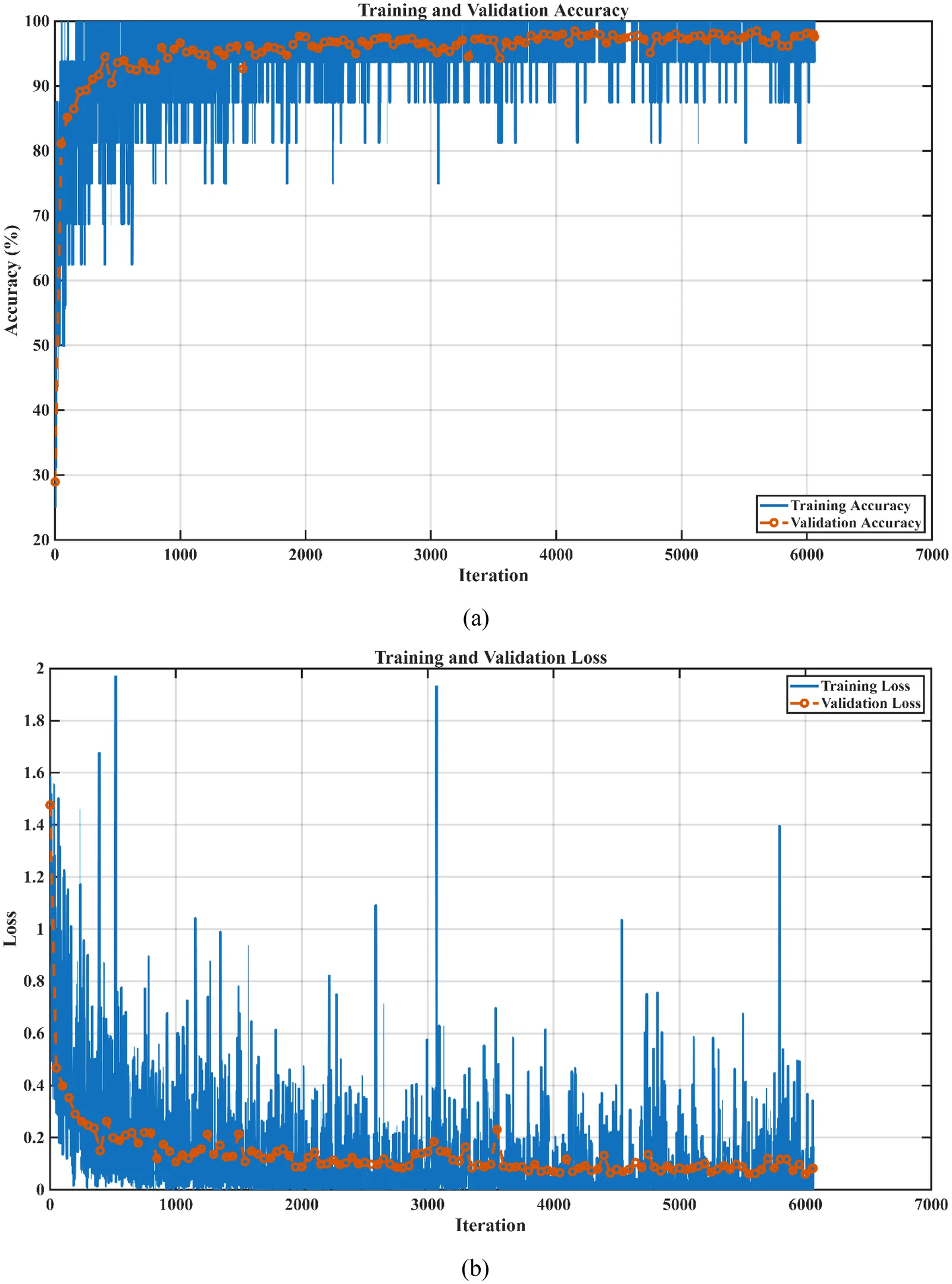
Training performance of the proposed model: **(a)** accuracy **(b)** loss curves.

The training and validation accuracy and loss curve of the proposed classifier is presented in **Figure 8**. It has been observed that the convergence is constant across the curves, and the validation accuracy is very similar to the training accuracy, thus, showing that there is minimal overfitting. The success of transfer learning in the acceleration of optimization can be supported by the rapid convergence that occurs after the initial epochs.

**Table 6** represents the precision, recall and F1-score of the proposed framework per class on the test set. The no-tumor class achieved perfect precision and recall, indicating zero false positives and false negatives. The classes glioma and meningioma had slightly lower performance which can be due to the overlapping of visual appearances whereas the pituitary tumor were diagnosed with very high sensitivity.

**Table 6:** The performance measures of the proposed framework on the test set.

Class	Precision (%)	Recall (%)	F1-score (%)
Glioma	99.3	98.0	98.6
Meningioma	98.0	97.7	97.8
No-tumor	100.0	100.0	100.0
Pituitary	98.7	99.7	99.2

**Figure 9:**
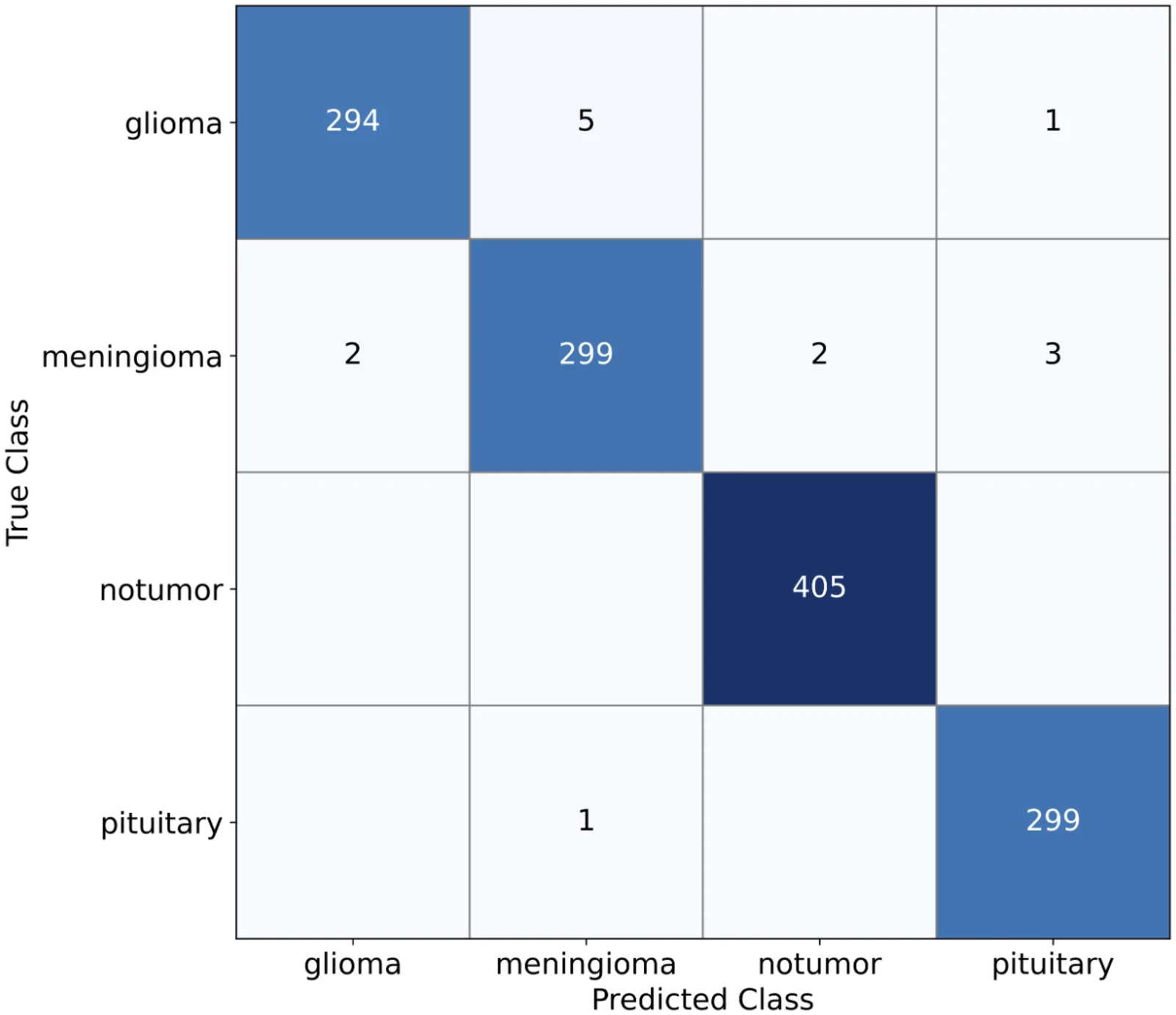
Confusion matrix of the proposed method.

The confusion matrix of the DAE-enhanced ResNet-50 framework is shown in **Figure 9**. The matrix shows that most predictions lie on the diagonal, indicating balanced classification across all classes. The confusion between glioma and meningioma classes is minimal, but it is also in line with the known radiological similarities (Cheng et al., 2015). Notably, there is no misclassification in the no-tumor class.

**Figure 10:**
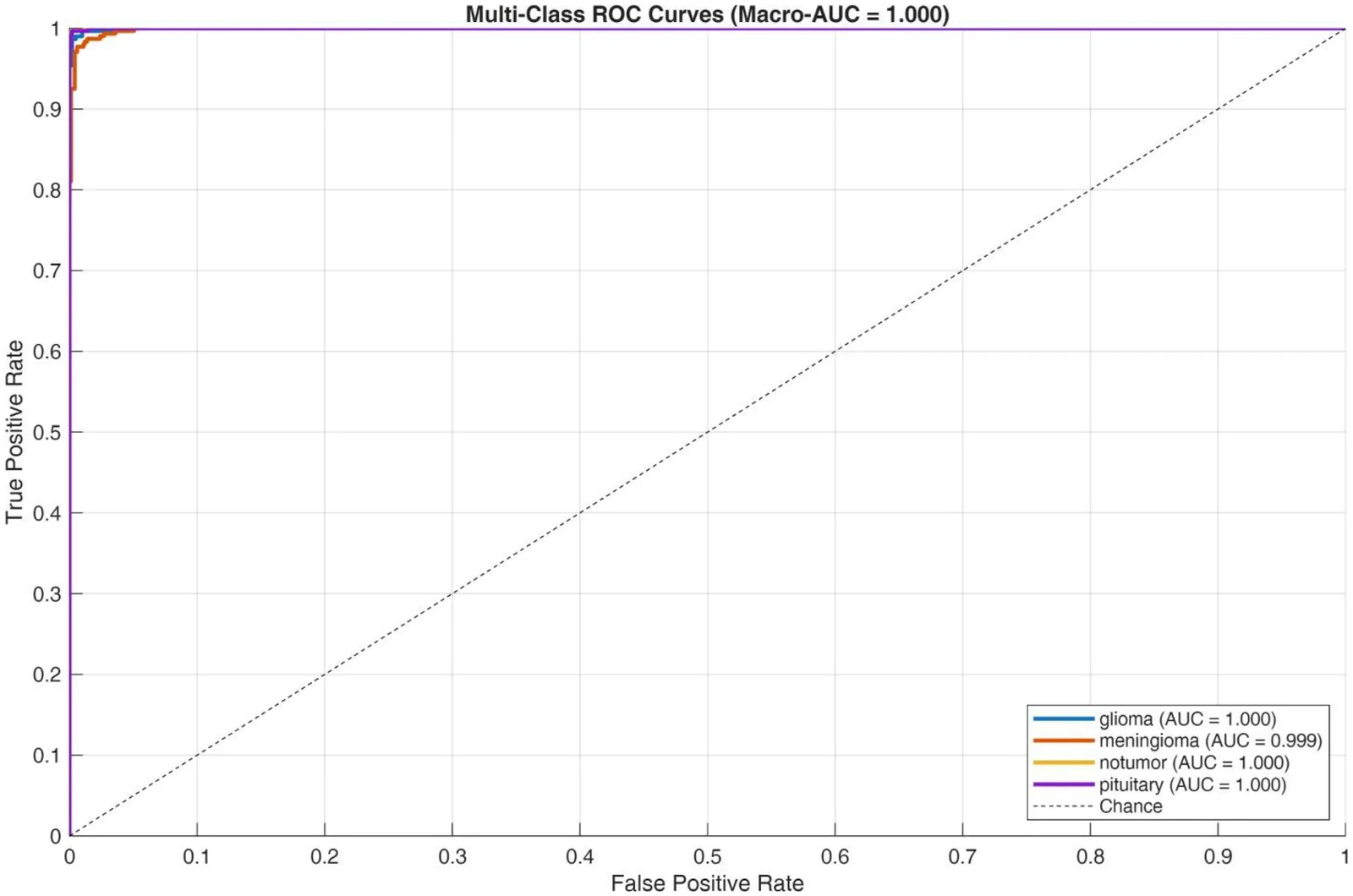
One-versus-rest ROC curves of the proposed model.

The multiclass ROC curves of the proposed framework using a one-vs-rest strategy are plotted in **Figure 10**. The ROC analysis indicates that there is almost a perfect discriminative performance in all classes. The framework achieved area under the ROC curve (AUC) values of 1.000 in glioma, no-tumor and pituitary tumor classes and 0.999 in the meningioma class with a macro-average AUC of 1.000. These results indicate excellent separability between tumor and non-tumor cases and among tumor subtypes.

### 4.1 Grad-CAM explainability results

**Figure 11:**
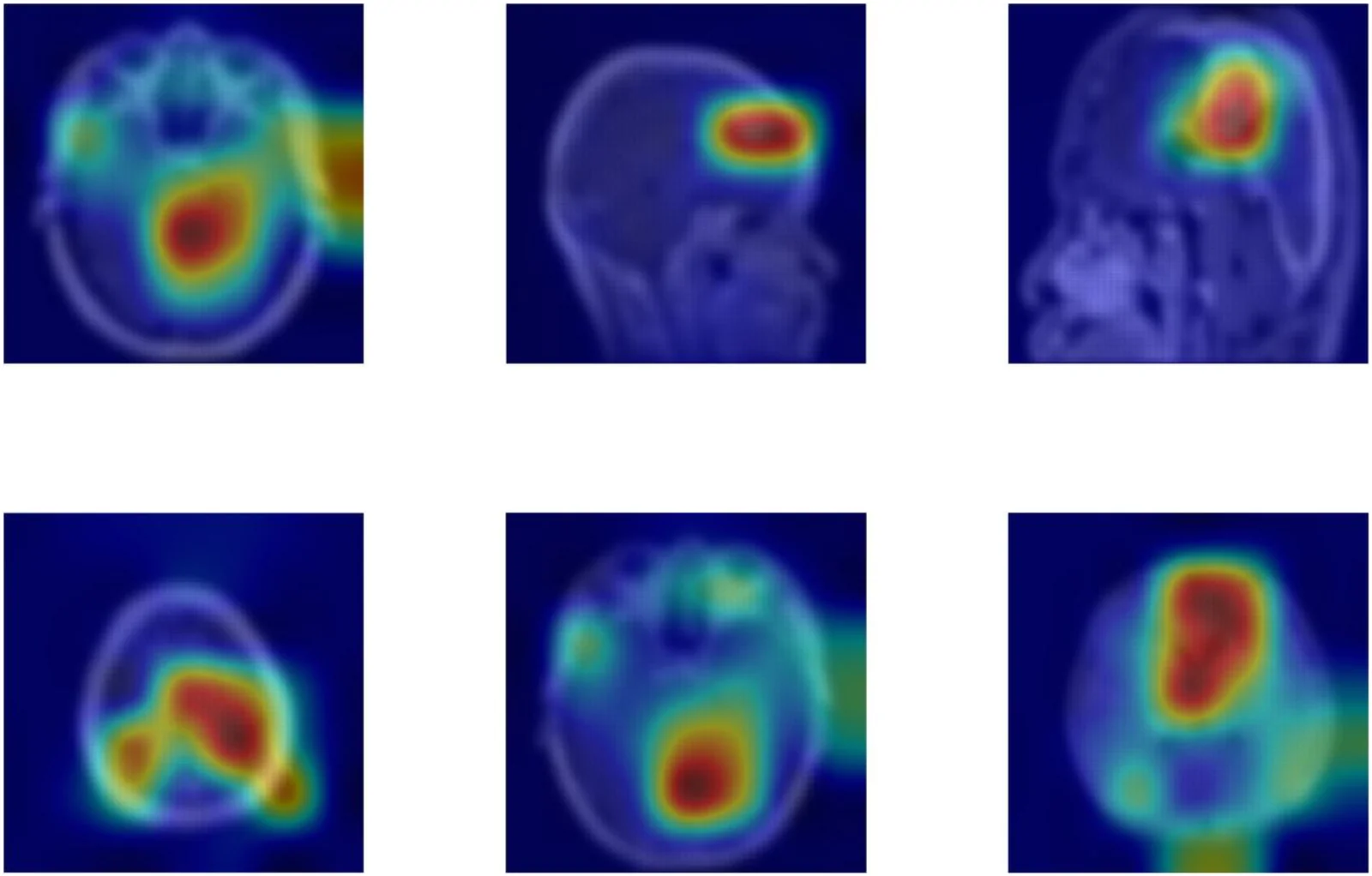
Grad-CAM visualizations for representative MRI test images.

The visualizations of Grad-CAM are shown in **Figure 11** and were created using test MRI images of various tumor classes. Grad-CAM highlights spatial regions within an input image that contribute most significantly to the model’s predicted class by back-propagating gradients from the final convolutional layer to the input space.

In the presented visualizations, warmer colors (red and yellow) indicate regions with a high contribution to the model’s decision, whereas cooler colors (blue) represent areas with minimal influence. As observed in **Figure 11**, the proposed DAE-enhanced ResNet-50 model consistently focuses on anatomically relevant image regions associated with tumor structures types and imaging planes. For glioma cases, the Grad-CAM maps emphasize irregular, infiltrative regions within the brain parenchyma, aligning with the known diffuse growth patterns of gliomas. In meningioma cases, the highlighted regions appear as relatively well-defined and localized areas near the brain surface, consistent with the extra-axial nature of meningiomas. Similarly, pituitary tumor visualizations demonstrate strong activation around the sellar and suprasellar regions, which is clinically relevant for pituitary adenomas. In no-tumor cases, the absence of intense localized activations confirms that the model does not rely on spurious patterns and instead learns discriminative tumor-specific features. Importantly, the Grad-CAM maps show minimal activation in irrelevant anatomical regions such as the skull, background, or non-tumorous brain tissue. This behavior indicates that the model’s predictions are driven by pathologically relevant structures rather than global intensity patterns or dataset biases. The effectiveness of Grad-CAM is further enhanced by the learned image enhancement stage using the denoising autoencoder, which improves contrast and suppresses noise, allowing clearer localization of salient tumor regions.

The Grad-CAM visualizations demonstrates that the proposed framework does not rely on irrelevant image regions such as the skull boundary, image corners, background artifacts, or non-pathological tissues during prediction. Instead, the activation maps remain spatially concentrated around diagnostically relevant tumor structures. It shows that the network learns discriminative image representations associated with tumor morphology and tissue heterogeneity. This behavior is particularly important in medical imaging applications as deep learning models may otherwise produce high classification accuracy while relying on dataset-specific biases or non-diagnostic image features.

Furthermore, the observed activation distributions are consistent with known radiological characteristics of different brain tumor subtypes. Glioma cases exhibit broader and irregular activation patterns corresponding to infiltrative tumor growth behavior. The meningioma cases show more compact and peripheral attention regions that align with their extra-axial anatomical presentation. Pituitary tumor activations are concentrated around sellar-region structures and it demonstrates anatomically coherent localization behavior. These findings are suggesting that the proposed framework captures image patterns associated with tumor morphology rather than relying solely on global intensity variations.

The explainability analysis also shows that the denoising autoencoder contributes not only to classification performance improvement but also to more stable and visually interpretable localization maps. By suppressing acquisition noise and enhancing structural contrast prior to feature extraction, the DAE facilitates clearer discrimination of tumor boundaries and improves the spatial consistency of Grad-CAM heatmaps across different MRI samples.

Although Grad-CAM provides useful visual interpretation of CNN decision-making, it remains a *post-hoc* explainability method and does not establish direct causal reasoning. Therefore, Grad-CAM results should be interpreted as supportive visual evidence rather than definitive clinical explanations. Nevertheless, the presented visualizations provide important transparency regarding model behavior and improve the transparency of the proposed framework for potential decision-support applications.

Overall, the Grad-CAM analysis shows that the proposed framework not only achieves strong quantitative performance but also it provides visually interpretable explanations of its predictions. The activation maps improve transparency regarding model behavior. It can support future investigation of explainable AI-assisted decision-support systems for brain tumor MRI analysis.

### 4.2 Cross-validation and statistical robustness analysis

To further evaluate the statistical reliability, reproducibility and generalization capability of the proposed Denoising Autoencoder–Enhanced ResNet-50 (DAE–ResNet50) framework, an additional five-fold stratified cross-validation analysis is conducted using the complete MRI dataset comprising 7023 images. Unlike the primary independent testing protocol, the cross-validation analysis is providing a more rigorous repeated-evaluation framework by exposing the proposed model to multiple unseen testing partitions while maintaining class distribution consistency across all folds. In the implemented algorithm, the complete dataset is divided into five mutually exclusive folds using stratified partitioning. During each fold, four folds were utilized for model development, while the remaining fold was reserved for testing. Furthermore, to avoid any possibility of information leakage, the denoising autoencoder (DAE) is independently trained using only the corresponding fold-specific training subset. The fold-trained DAE is subsequently employed to enhance the MRI images of the associated training, validation, and testing subsets before classification using the modified ResNet-50 architecture. This procedure ensures a statistically rigorous and leakage-resistant evaluation methodology.

**Figure 12:**
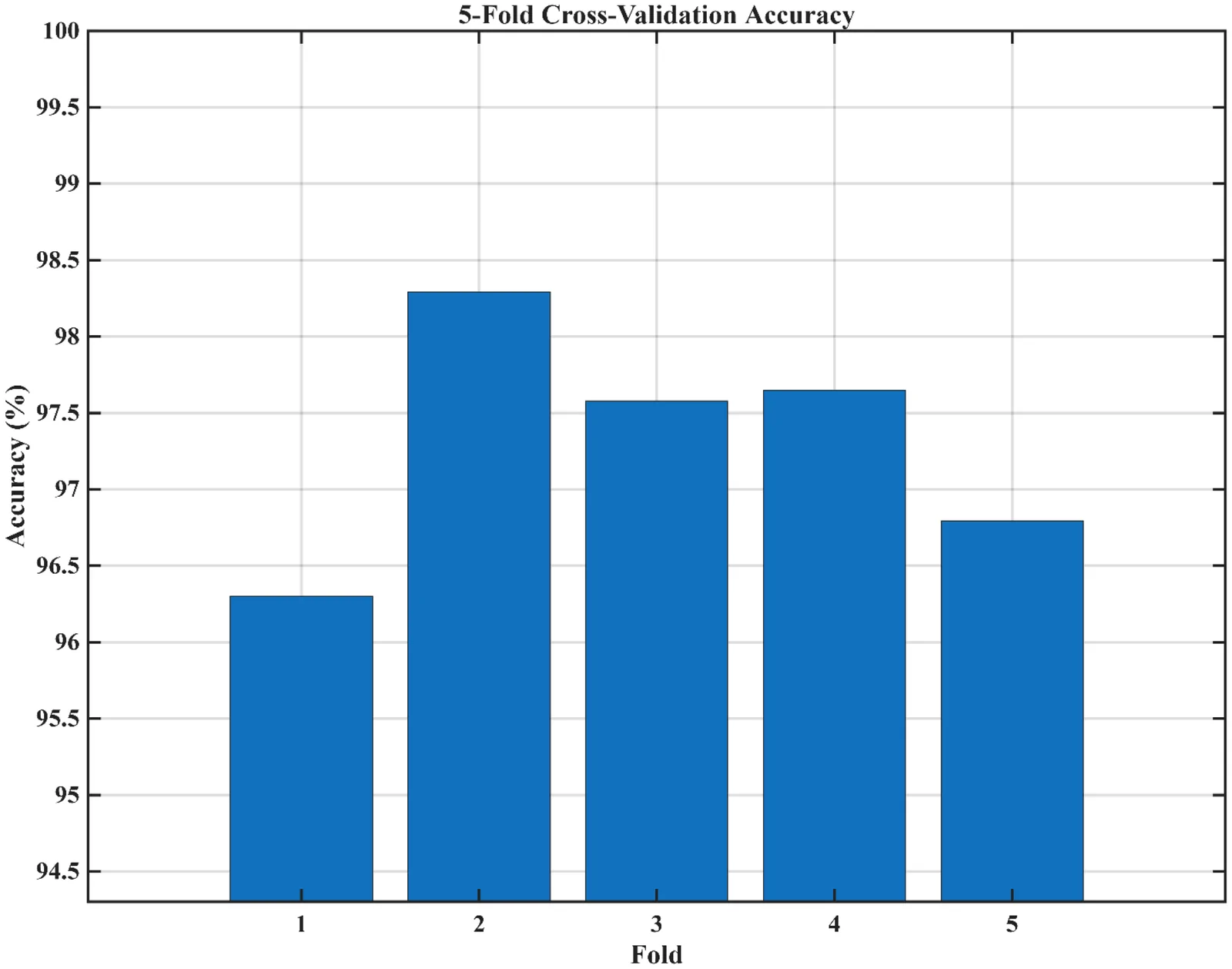
Five-fold cross-validation accuracy results.

The obtained cross-validation results show highly stable and consistent performance across all folds. **Figure 12** shows the fold-wise classification accuracy achieved by the proposed framework during the five-fold evaluation process. The obtained accuracies remained consistently high across all folds which is confirming strong generalization capability under repeated unseen testing conditions.

**Figure 13:**
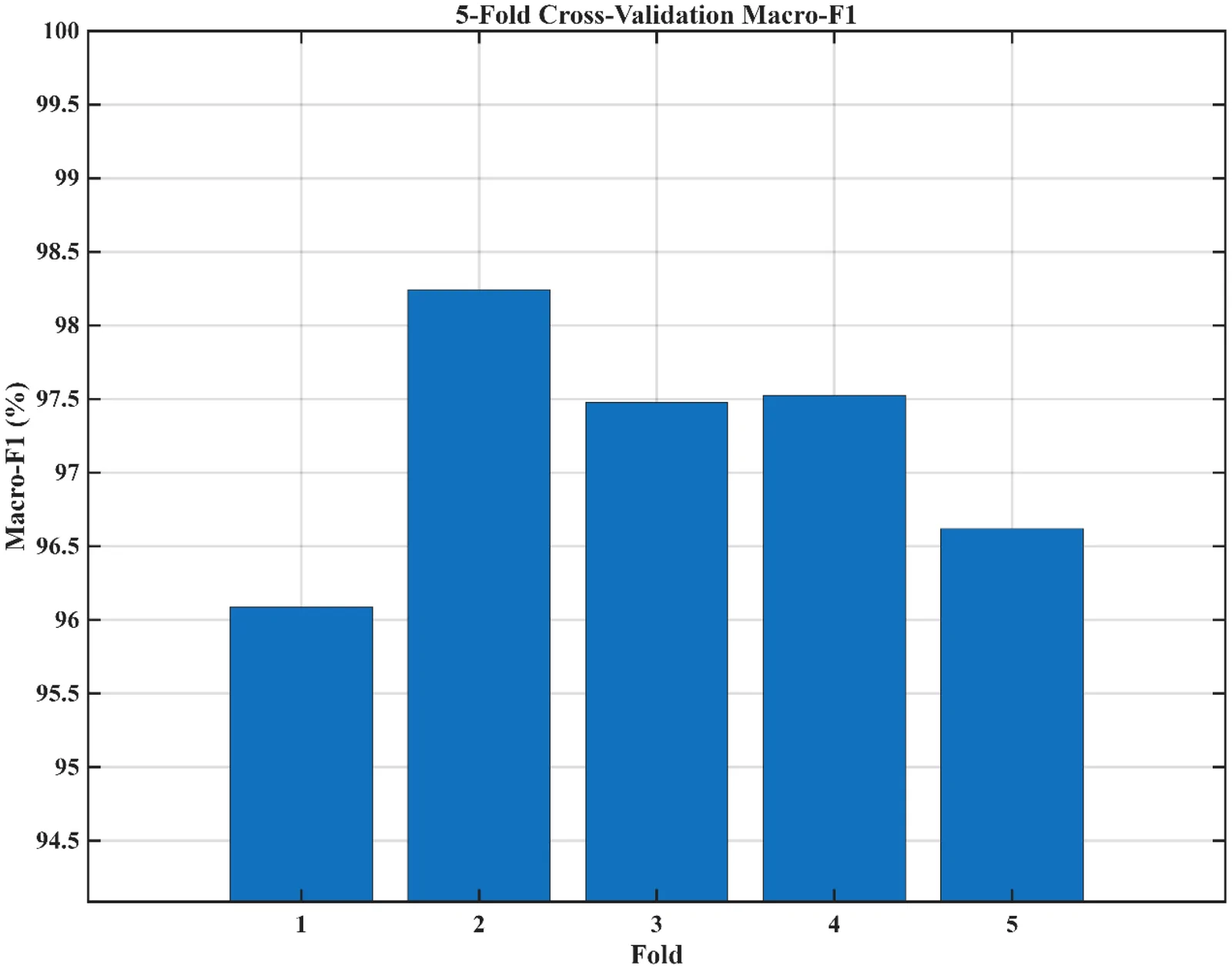
Five-fold cross-validation macro-F1 score results.

Similarly, **Figure 13** shows the macro-F1 scores obtained during the five-fold validation analysis. The proposed framework is achieving highly consistent macro-F1 performance across different fold partitions. This is indicating balanced precision–recall behavior for all tumor categories.

**Figure 14:**
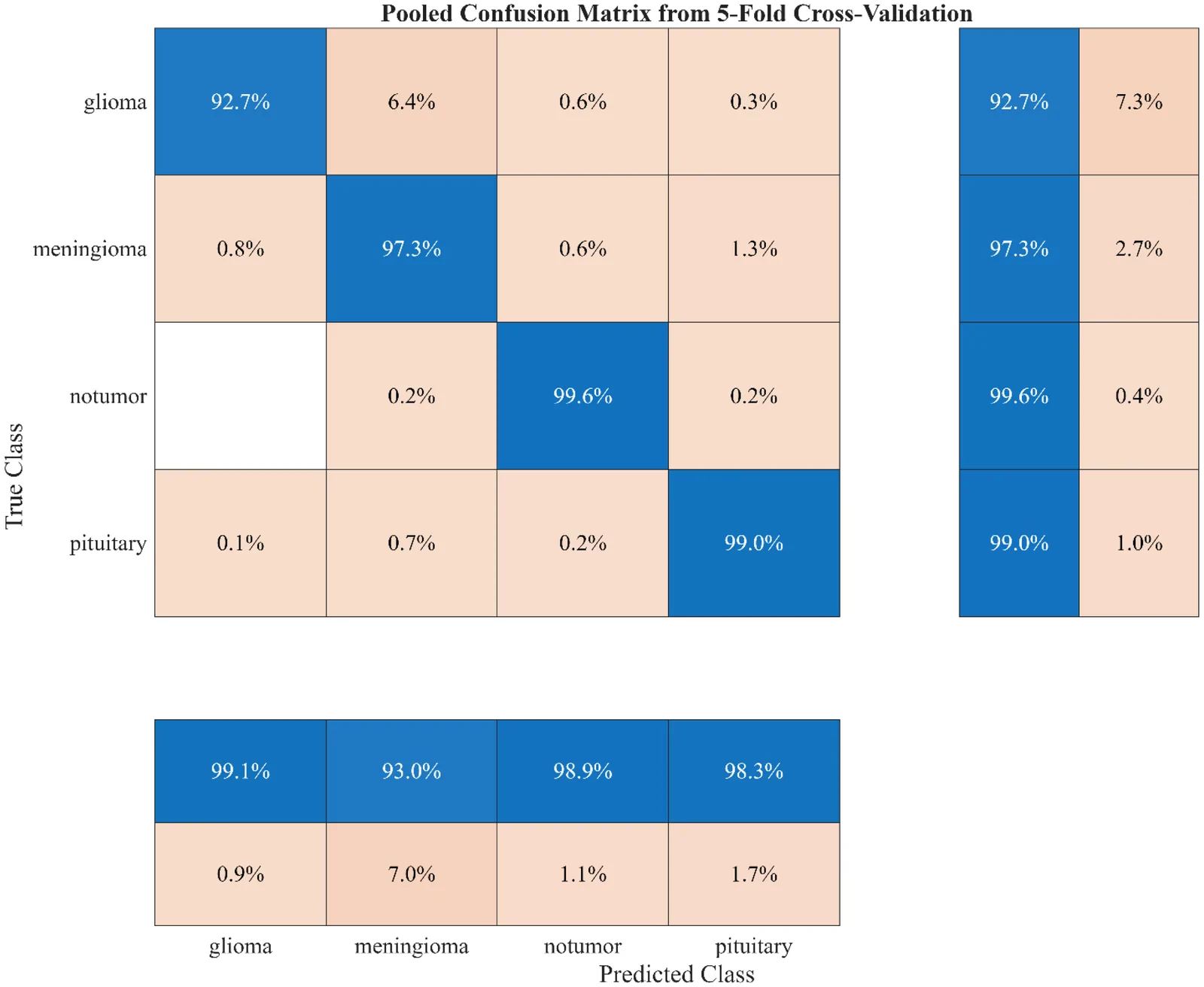
Pooled confusion matrix from five-fold cross-validation.

The fold-wise experimental results give accuracies of 96.30%, 98.29%, 97.58%, 97.65%, and 96.80%, respectively. This is resulting in an average classification accuracy of 97.32% with a standard deviation of only ±0.78%. In addition, the proposed framework is achieving an average macro-F1 score of 97.19% ± 0.84%, which is further confirming the robustness and reproducibility of the proposed methodology. To further investigate class-wise generalization capability, the pooled confusion matrix which is obtained from all five folds is shown in **Figure 14**. The confusion matrix gives strong discrimination performance across all tumor categories, with only minor misclassification observed between visually similar classes such as glioma and meningioma. The no-tumor and pituitary tumor categories give particularly high classification consistency across all folds.

**Figure 15:**
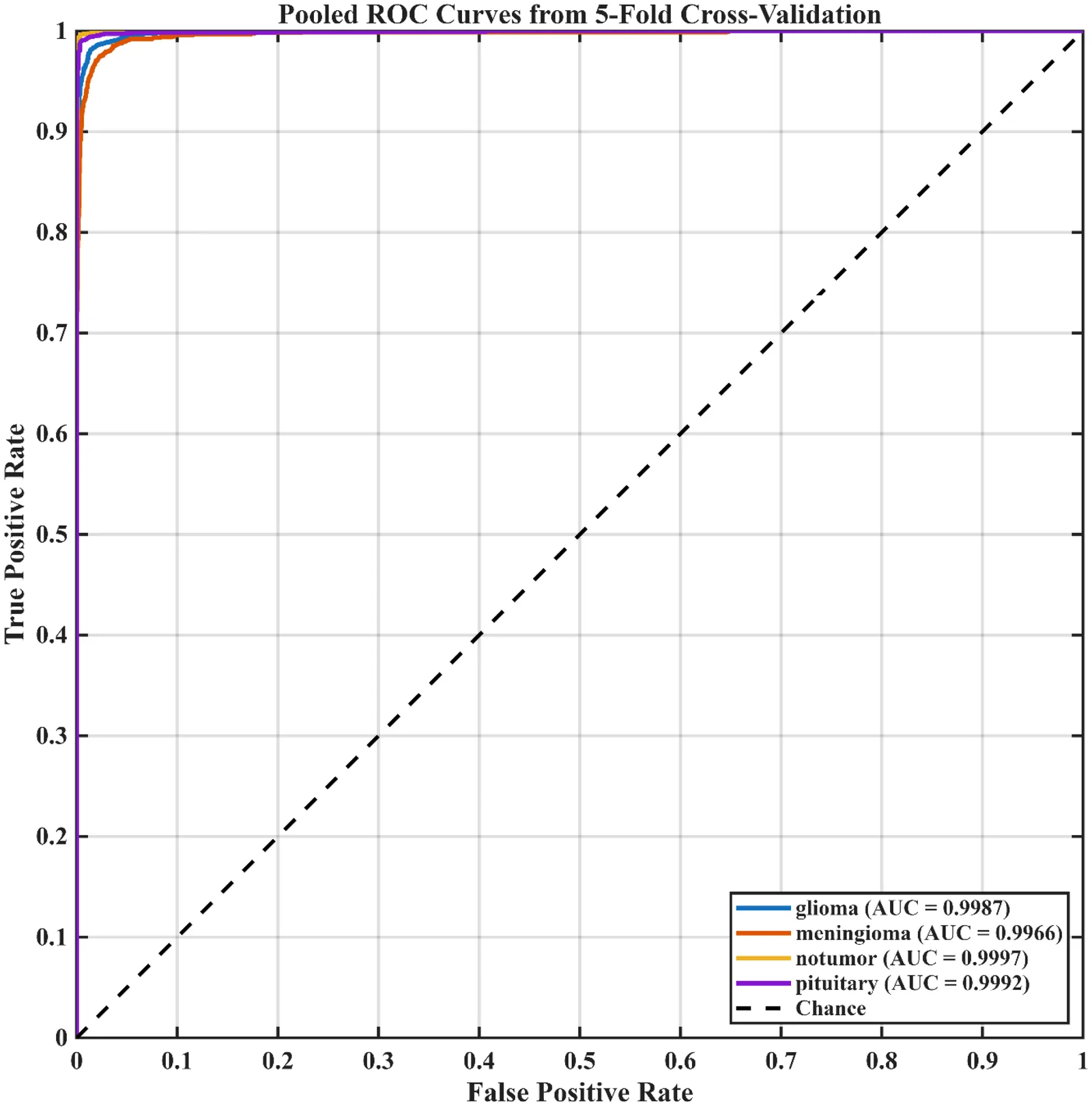
Pooled one-versus-rest ROC curves from five-fold cross-validation.

Furthermore, the pooled one-versus-rest ROC curves which are obtained from the five-fold evaluation is illustrated in **Figure 15**. The proposed framework has achieved extremely high discriminative capability for all classes, with macro-AUC values consistently approaching unity across all folds. Specifically, the obtained macro-AUC values ranged between 0.99839 and 0.99926, which is indicating excellent separability among tumor categories even under repeated evaluation conditions.

A detailed statistical summary of the five-fold cross-validation analysis has been provided in **Table 7**. This table is reporting the mean, standard deviation (SD), standard error of mean (SEM), and corresponding 95% confidence intervals for all major performance metrics. The results shows that the proposed framework is maintaining highly stable performance with narrow confidence intervals. Thus it is validating the statistical robustness of the developed model.

**Table 7:** Statistical summary of five-fold cross-validation results including mean, standard deviation (SD), standard error of mean (SEM), and 95% confidence intervals.

Metric	Mean	SD	SEM	95% CI lower	95% CI upper
Accuracy (%)	97.32	0.78	0.35	96.36	98.29
Macro-Precision (%)	97.34	0.76	0.34	96.40	98.28
Macro-Recall (%)	97.16	0.84	0.38	96.11	98.21
Macro-F1 (%)	97.19	0.84	0.38	96.14	98.24
Macro-AUC	0.99885	0.00036	0.00016	0.99840	0.99929

The obtained statistical results have further revealed a mean macro-precision of 97.34%, mean macro-recall of 97.16%, and mean macro-AUC of 0.99885. Moreover, Wilson confidence interval analysis is performed to further quantify the reliability of the obtained classification accuracy. The pooled Wilson 95% confidence interval for classification accuracy is computed as 96.92%–97.68%, which is confirming the robustness and repeatability of the proposed framework under multiple unseen testing conditions.

Overall, the additional five-fold cross-validation and confidence interval analyses substantially strengthen the statistical validity of the proposed DAE–ResNet50 framework and significantly reduces concerns regarding overfitting, dataset dependency, and performance instability. The obtained results are confirming that the proposed framework maintains strong and reproducible classification performance under repeated evaluation scenarios. This is supporting its suitability for intelligent MRI-based brain tumor classification applications.

### 4.3 Comparison with other models

#### 4.3.1 Comparison of the proposed model with CNN and transformer-based architectures

To further evaluate the effectiveness and generalization capability of the proposed DAE-enhanced ResNet-50 framework, additional comparative experiments are conducted using several alternative deep learning architectures, including DenseNet-201, EfficientNet-B0, a U-Net-style classifier, MobileNetV2, and a Vision Transformer (ViT-Tiny-16). All models were trained and evaluated using the same dataset partitioning strategy and experimental settings to ensure a fair comparison. The experimental results demonstrate that the proposed DAE + ResNet-50 framework has achieved a classification accuracy of 98.93%, which is confirming its strong capability for multi-class brain tumor MRI classification. Among the alternative CNN-based architectures, EfficientNet-B0 achieved 98.78% accuracy, and DenseNet-201 has achieved 97.94% accuracy. Although both architectures are giving competitive results, however, they remained slightly below the performance of the proposed framework. MobileNetV2, representing a lightweight CNN architecture, has achieved 96.26% accuracy, which is indicating that computationally efficient networks may experience reduced discriminative capability when addressing fine-grained tumor classification tasks.

To further strengthen the comparative analysis with transformer-based models, a ViT-Tiny-16 architecture was also evaluated. The DAE + ViT-Tiny-16 framework achieved 92.07% accuracy. This observation suggests that, for the utilized dataset size and characteristics, convolutional feature extraction remains more effective than lightweight transformer representations.

In contrast, the U-Net-style classifier exhibited substantially lower classification performance (66.51% accuracy), indicating that architectures originally designed for image segmentation are less suitable for direct multi-class classification tasks without dedicated classification-oriented modifications.

**Figure 16:**
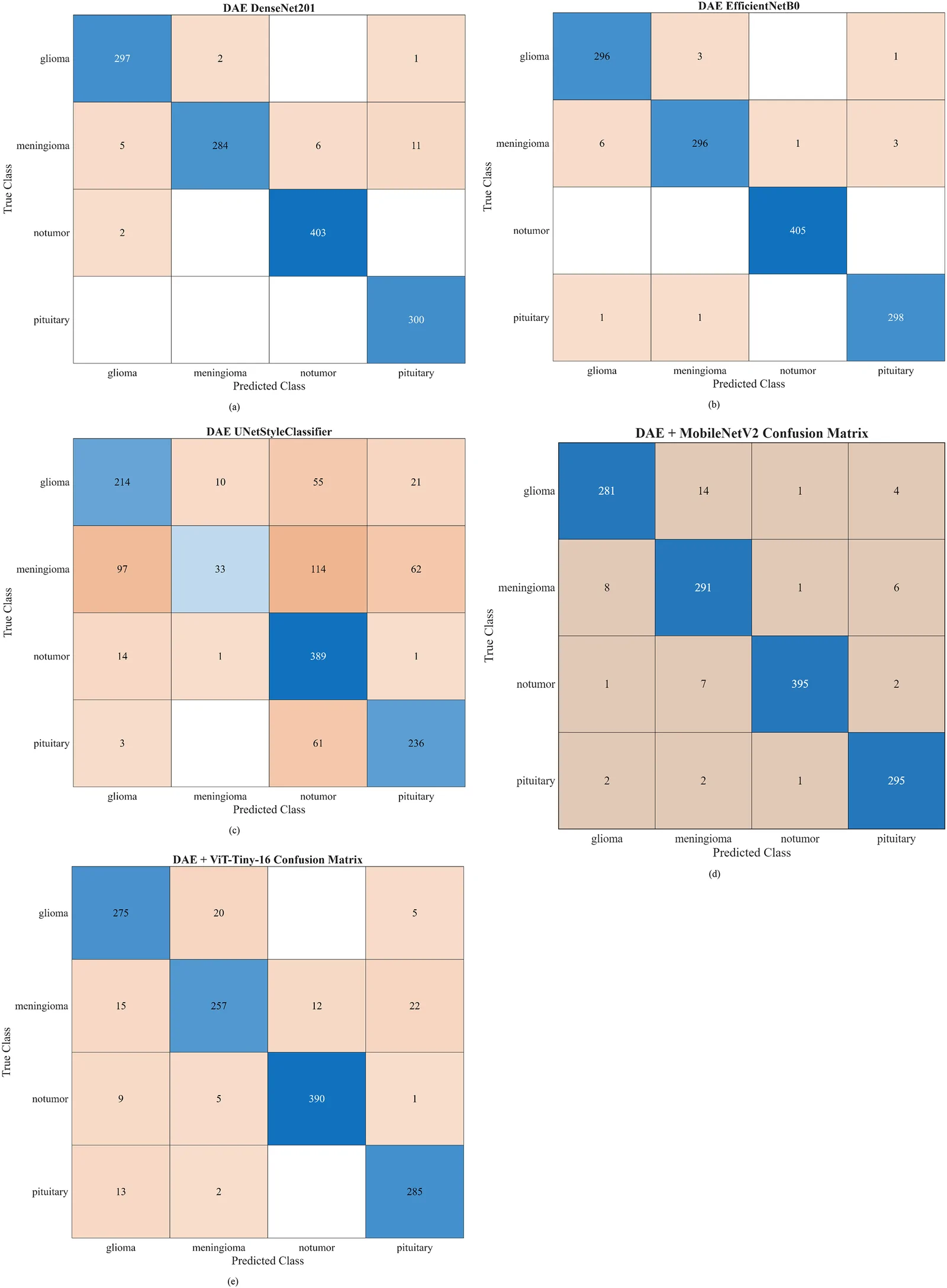
The comparative confusion matrices of **(a)** DenseNet-201, **(b)** EfficientNet-B0 **(c)** U-Net **(d)** Mobilenetv2 **(e)** ViT -Tiny.

Overall, the results show that the proposed DAE-enhanced ResNet-50 framework consistently provides the best balance between classification accuracy, feature discrimination capability, and robustness among all evaluated CNN-based and transformer-based architectures. The comparative confusion matrices are presented in **Figure 16**.

#### 4.3.2 Comparison of GradCAM with LIME and SHAP

**Figure 17:**
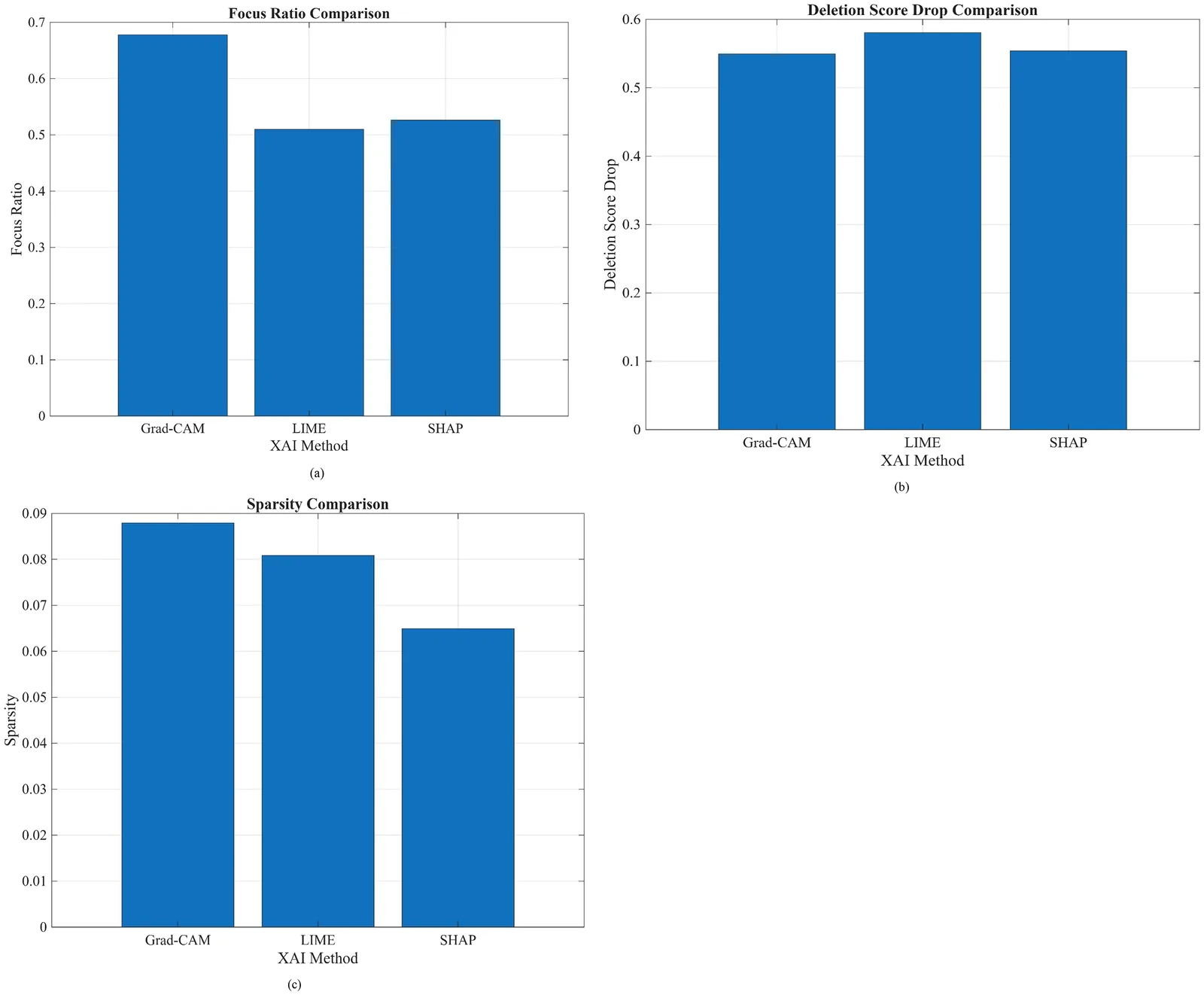
Quantitative comparaison for XAI Methods **(a)** Focus Ratio Comparison **(b)** Deletion Score Drop comparaison **(c)** Sparsity comparison.

**Figure 18:**
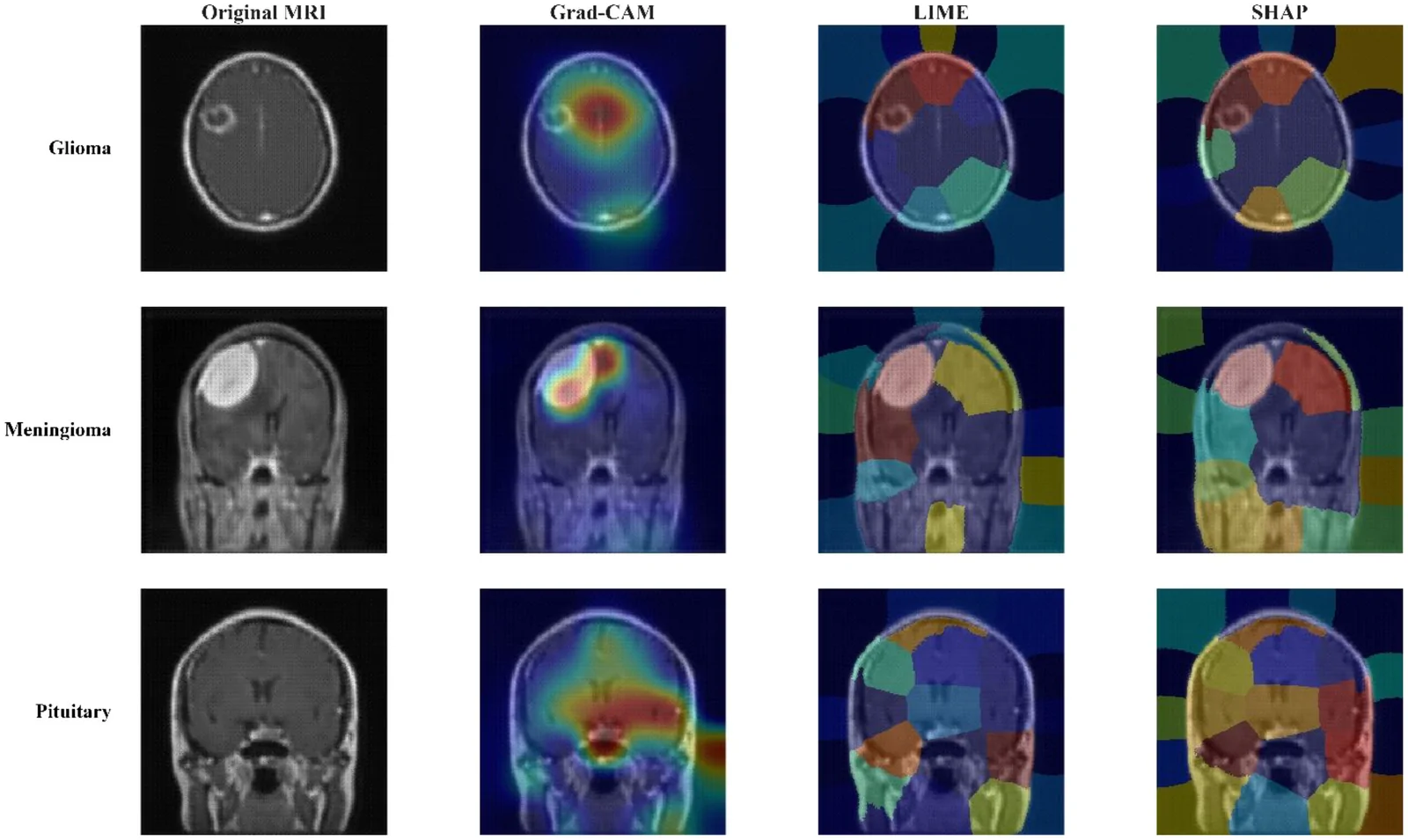
Qualitative visual comparisons between Grad-CAM, LIME, and SHAP activation maps.

The experiments are conducted for the quantitative XAI evaluation framework based on multiple explainability metrics, including Focus Ratio, Deletion Score Drop and Sparsity analysis. Comparative quantitative results are summarized in the **Table 8** and also shown in **Figure 17**. In addition, qualitative visual comparisons between Grad-CAM, LIME, and SHAP activation maps are shown in **Figure 18**. Experimental results show that Grad-CAM achieved the highest Focus Ratio (0.678). This is indicating superior spatial localization and stronger concentration of attention within clinically relevant tumor regions. Although LIME achieved a slightly higher deletion score drop, however, the Grad-CAM produced substantially more spatially coherent and anatomically meaningful activation maps. SHAP gives moderate localization performance but generated comparatively more diffuse activation patterns. The visual comparison results further show that Grad-CAM consistently highlighted compact and clinically interpretable tumor regions. On the other hand, LIME and SHAP often producing fragmented or spatially scattered explanations across non-tumor regions. These findings indicate that Grad-CAM provides a more suitable balance between localization capability, interpretability, and computational efficiency for CNN-based brain tumor MRI classification.

**Table 8:** XAI Summary for Grad-CAM, LIME, and SHAP.

XAI Method	Group Count	Mean Focus Ratio	Mean Deletion Score Drop	Mean Sparsity
Grad-CAM	24	0.6778438	0.549559086561203	0.08789685
LIME	24	0.5099438	0.580662888785203	0.08081242
SHAP	24	0.5261866	0.553887270390987	0.06492012

### 4.4 Comparison with existing studies

The proposed DAE-enhanced ResNet-50 framework is compared to the recent peer-reviewed studies on brain tumor MRI classification that have been published in 2020–2025 (14–19, 32–35). The performance comparison with published papers has been summarized in **Table 9**.

**Table 9:** Comparison of the proposed method with published studies.

Ref.	Study	Method	Tumor Classes	Accuracy (%)	Explainability
(14)	Zahoor et al. (2024)	Transfer learning CNN	3	98.2	No
(15)	Gómez-Guzmán et al. (2025)	Transfer learning (CNN backbones)	4	98.17	No
(16)	Shoaib et al. (2025)	Pretrained CNNs + ML classifiers	4	98.0	No
(19)	Disci et al. (2025)	Transfer learning (pretrained CNNs)	4	98.73	Not emphasized
(32)	Kumar et al. (2020)	Optimization-driven CNN	4	96.3	No
(33)	Rasheed et al. (2023)	Image enhancement + CNN	4	97.84	No
(34)	Noreen et al. (2021)	Fine-tuned CNN + ensemble	4	94.34	No
(35)	Masood et al. (2021)	Mask Region-based Convolution neural network	4	98.34	No
–	Proposed method	DAE + ResNet-50 + Grad-CAM	4	98.93	Yes (Grad-CAM)

Relative to recent CNN-based approaches including the Res-BRNet architecture suggested by Zahoor et al. (14), which reached a classification precision of 98.22 on publicly accessible brain MRI datasets, the framework discussed shows a higher degree of robustness and practical applicability to the use of architectural design alternatives. Although Res-BRNet is successful in using residual feature learning, regional feature learning to learn spatial tumor features, it uses raw or traditionally preprocessed MRI images and does not directly tackle the issue of noise reduction or acquisition variability. By comparison, the suggested approach encompasses a specific image-improving stage involving a denoising autoencoder before classification and boosts image quality and stabilizes feature extraction in the presence of noisy and heterogeneous imaging. Consequently, the suggested scheme achieves a more significant test accuracy of 98.93, and it also incorporates Grad-CAM-based explainability, thus providing a better diagnostic quality as well as explainable decision support that is applicable to real-world Digital Health implementation.

The proposed framework has better performance in comparison to hybrid CNN-Transformer schemes like that of Gomez-Guzman et al. (15) which recorded a 97.17% accuracy in multi-class brain tumor classification, which does not explicitly integrate learned image enhancement and explainability, which are all not provided in (15).

The proposed framework provides high-quality performance compared to hybrid CNN machine learning methods like Shoaib et al. (16) that 98 percent accuracy, which provides end-to-end optimization, learned image enhancement, and explicit explainability.

Another nearby recent research by Disci et al. (19) compared brain tumor MRI classification to multiple pretrained deep CNN models in a transfer learning architecture with a 98.73 accuracy. Although their findings indicate that this task can be performed successfully with the help of transfer learning, the method assumes a direct classification of MRI images without a specific learned image-enhancing or image-denoising step. On the contrary, the suggested structure incorporates a denoising autoencoder before classifications that enhances the resilience to MRI noise and acquisition variation. In addition to this, the proposed approach obtains a marginally better accuracy of 98.93% with an added offer of Grad-CAM-driven explainability, which facilitates more transparent and more interpretable decision-making.

The proposed framework corresponds to better robustness and model transparency compared to optimization-based CNN models like the one suggested by Kumar and Mankame (32), which achieved a high classification accuracy of 96.3 but did not explicitly suppress noise and explain the results with Grad-CAM.

The proposed method, in comparison to recent CNN-based methods including the image-enhancement-assisted framework developed by Rasheed et al. (33) with a classification accuracy of 97.84 per cent on a four-class brain tumor MRI dataset, performs better besides the addition of a specific denoising autoencoder stage to remove noise. In contrast to (33) which depends on traditional methods of enhancement (Gaussian sharpening and CLAHE), the given framework uses learned feature-level denoising, leading to a higher resistance to acquisition noise and a higher ability to generalize to tumor categories.

The proposed framework is significantly better compared to the ensemble-based deep learning methods, such as the one suggested by Noreen et al. (34), where the classification rates of around 96–97 can be achieved with the help of fine-tuned convolutional neural networks, yet the architecture remains simple. Even though ensemble strategies enhance robustness through the combination of multiple models, they are also associated with higher computational costs and have no explicit noise suppression and interpretation mechanisms. Conversely, the introduced denoising autoencoder-modified ResNet-50 system has a better accuracy of 98.93% and directly encourages the learned image enhancement and Grad-CAM-based explainability, which makes it more suitable for future AI-assisted decision-support applications.

The proposed framework provides a better classification-based architecture with an accuracy of 98.93 as compared to the region-based deep learning models like the Mask R-CNN that used the DenseNet-41 architecture reaching an accuracy of 98.34. More to the point, the proposed approach combines a denoising autoencoder to improve the quality of MRI images before classification and uses Grad-CAM-based explainability, which contributes to the increased resistance to acquisition noise and model transparency, unlike (35).

Although these methods show high levels of discriminative performance, the majority of them are mainly interested in the accuracy of classification but they do not explicitly present learned image enhancement, or explainability mechanisms as essential parts of the framework. Conversely, the proposed approach combines image enhancement based on denoising autoencoders, deep transfer learning with ResNet-50 and explainability based on Grad-Cam into a single pipeline. The design permits better noise sensitivity and variability of acquisitions and offers clear visual descriptions that are consistent with clinical thinking. Consequently, the suggested framework has higher classification performance and addresses important requirements for explainable and robust Digital Health research systems.

### 4.5 Ablation study

In order to assess the role of each of the individual components in the proposed framework, an ablation experiment is performed by removing or altering individual modules systematically and noting the resulting changes in performance. The ablation experiment is aimed at estimating the effects of the denoising autoencoder (DAE) and transfer-learning approach as used by the ResNet- 50 classifier. Direct training of the ResNet-50 classifier on the processed MRI images without the DAE augmentation phase give a significant loss in performance. This setup show a higher false classification between glioma and meningioma classes as it less resistant to noise and changes in intensity. Traditional preprocessing methods have only offered slight improvements. But they did not match the performance offered by the learned enhancement method. Inclusion of the DAE results in increased feature clarity and better structural representation, which resulted in increased accuracy and macro-F1 score. The results of these experiments are indicating that data-based denoising is important to enhance the classification robustness and discriminative capability. **Table 10** provides a study of ablation analyzing the effect of the denoising autoencoder.

**Table 10:** Summary of the ablation study evaluating the impact of the denoising autoencoder.

Configuration	Accuracy (%)	Macro-F1 (%)
ResNet-50 without DAE	96.8	96.5
ResNet-50 + conventional preprocessing	97.6	97.2
Proposed DAE + ResNet-50	98.93	98.7

In order to evaluate the significance of transfer learning, another set up was tested where a shallow CNN was trained on the same dataset only. This setup had lower convergence and worse generalization. This shows the value of using pretrained weight which are trained on large datasets (Shin et al., 2016). Overall, the ablation experiment are proving the statements that the denoising autoencoder and the transfer-learning-based ResNet-50 architecture are the key elements of the proposed framework. Although the proposed framework demonstrated strong performance on a publicly available benchmark dataset, the current study does not include external clinical validation, multi-center evaluation, or comparison with expert radiologists. Consequently, the presented results should be interpreted as evidence of methodological feasibility rather than immediate clinical readiness. Future work will investigate independent clinical datasets and expert-assisted validation to further assess real-world applicability.

## 5 Conclusion

This paper introduces a robust, explainable deep-learning model of multi-classification of brain tumor based on MRI scans. The proposed method incorporating a denoising autoencoder-based denoising step and a transfer-learning-based ResNet-50 classifier is an effective solution to major issues of noise, variability, and interpretability in medical imaging. The experimental findings show that the given framework was able to reach a test accuracy of 98.93%, and the class-wise high scores of precision, recall, and F1-score. The no-tumor class was observed to have an ideal classification and the tumor classes had strong discriminative behavior. The ROC analysis also validated almost perfect class separability with a macro-average AUC of 1.000. In addition to quantitative performance, incorporation of Grad-CAM provided visually interpretable explanations by highlighting image regions that contributed most strongly to the classification decisions. Such focus on explainability aligns with current recommendations for transparent and interpretable AI systems in medical imaging and may facilitate future clinical acceptance. Overall, the proposed framework provides a promising and interpretable approach for automated brain tumor MRI classification. Although the obtained results demonstrate strong classification performance and consistent explainability behavior, further validation using independent multi-center datasets and comparison with expert radiologists are required before clinical deployment. The framework may serve as a foundation for future AI-assisted decision-support systems in neuro-oncology.

## Data Availability

Publicly available datasets were analyzed in this study. This data can be found here: https://www.kaggle.com/datasets/masoudnickparvar/brain-tumor-mri-dataset.
