# Supplementary material for: A denoising autoencoder–enhanced ResNet 50 framework for explainable brain tumor classification using MRI
Source: Front Oncol. 2026 Aug 14;16:1806663. doi: 10.3389/fonc.2026.1806663 (PMC13522818; doi:10.3389/fonc.2026.1806663)
Supplement: Supplementary file 1 [file DataSheet1.pdf]

Supplementary Material

Algorithm 1. Pseudocode-based workflow of the proposed DAE-enhanced ResNet-50 framework

Input:

Brain MRI dataset D with four classes:

glioma, meningioma, pituitary tumor, and no-tumor

Output:

Predicted tumor class, classification performance metrics,

and explainability maps using Grad-CAM, LIME, and SHAP

Step 1: Load Dataset

Load MRI images from predefined Training and Testing folders

Assign class labels based on folder names

Split training data into training and validation subsets

Step 2: Preprocess MRI Images

Resize all MRI images to 224 × 224 pixels

Convert grayscale images to three-channel format if required

Normalize pixel intensity values to the range [0, 1]

Apply data augmentation only to the training subset

Step 3: Train Denoising Autoencoder

Add synthetic noise to training MRI images

Feed noisy images into the DAE encoder

Learn compact latent feature representation

Reconstruct denoised MRI images using the DAE decoder

Optimize the DAE using mean squared error loss

Step 4: Generate Enhanced MRI Images

Pass training, validation, and testing images through the trained DAE

Obtain denoised/enhanced MRI images

Use enhanced images as inputs to the classifier

Step 5: Modify ResNet-50 Classifier

Load pretrained ResNet-50 backbone

Replace final fully connected layer with a four-neuron layer

Replace the classification layer for four-class tumor classification

Set optimizer, learning rate, batch size, and number of epochs

Step 6: Train Classifier

Train the modified ResNet-50 using enhanced training images

Monitor validation accuracy and validation loss

Save the best-performing trained model

Step 7: Evaluate Classification Performance

Predict labels for the independent testing set

Compute accuracy, precision, recall, F1-score, and macro-AUC

Generate confusion matrix and ROC curves

Step 8: Compare with Other Models

Train and evaluate DenseNet-201, EfficientNet-B0,

and U-Net-style classifier using the same dataset split

Compare all models using accuracy, macro-F1, and macro-AUC

Step 9: Perform Explainability Analysis

Generate Grad-CAM heatmaps for representative test images

Generate LIME and SHAP explanation maps for comparison

Quantitatively evaluate XAI methods using Focus Ratio,

Deletion Score Drop, and Sparsity metrics

Step 10: Save Outputs

Save trained models, classification tables, XAI metrics,

confusion matrices, ROC curves, and high-resolution figures

Return:

Final classification results and explainability outputs

……………………………………………………………………………………………………….
